# Characterizing the marine mammal exposome by iceberg modeling, linking chemical analysis and *in vitro* bioassays†

**DOI:** 10.1039/d3em00033h

**Published:** 2023-05-03

**Authors:** Eva B. Reiter, Beate I. Escher, Elisa Rojo-Nieto, Hannah Nolte, Ursula Siebert, Annika Jahnke

**Affiliations:** a Department of Ecological Chemistry, Helmholtz Centre for Environmental Research – UFZ Permoserstr. 15 04318 Leipzig Germany annika.jahnke@ufz.de eva.reiter@ufz.de; b Department of Cell Toxicology, Helmholtz Centre for Environmental Research – UFZ Permoserstr. 15 04318 Leipzig Germany; c Environmental Toxicology, Department of Geosciences, Eberhard Karls University Tübingen Schnarrenbergstr. 94-96 72076 Tübingen Germany; d Institute for Environmental Research, RWTH Aachen University Aachen 52074 Germany; e Institute for Terrestrial and Aquatic Wildlife Research, University of Veterinary Medicine Hannover, Foundation Werftstr. 6 25761 Büsum Germany

## Abstract

The present study complements work on mixture effects measured with *in vitro* bioassays of passive equilibrium sampling extracts using the silicone polydimethylsiloxane (PDMS) in organs from marine mammals with chemical profiling. Blubber, liver, kidney and brain tissues of harbor porpoise (*Phocoena phocoena*), harbor seal (*Phoca vitulina*), ringed seal (*Phoca hispida*) and orca (*Orcinus orca*) from the North and Baltic Seas were investigated. We analyzed 117 chemicals including legacy and emerging contaminants using gas chromatography-high resolution mass spectrometry and quantified 70 of those chemicals in at least one sample. No systematic differences between the organs were found. Only for single compounds a clear distribution pattern was observed. For example, 4,4′-dichlorodiphenyltrichloroethane, enzacamene and etofenprox were mainly detected in blubber, whereas tonalide and the hexachlorocyclohexanes were more often found in liver. Furthermore, we compared the chemical profiling with the bioanalytical results using an iceberg mixture model, evaluating how much of the biological effect could be explained by the analyzed chemicals. The mixture effect predicted from the quantified chemical concentrations explained 0.014–83% of the aryl hydrocarbon receptor activating effect (AhR-CALUX), but less than 0.13% for the activation of the oxidative stress response (AREc32) and peroxisome-proliferator activated receptor (PPARγ). The quantified chemicals also explained between 0.044–45% of the cytotoxic effect measured with the AhR-CALUX. The largest fraction of the observed effect was explained for the orca, which was the individuum with the highest chemical burden. This study underlines that chemical analysis and bioassays are complementary to comprehensively characterize the mixture exposome of marine mammals.

## Abstract 2

The analysis of mixtures of environmental contaminants from marine mammal organs revealed distinct distribution patterns for single compounds. The combination of chemical analysis and bioassays can comprehensively characterize the mixture exposome.

## Graphical abstract

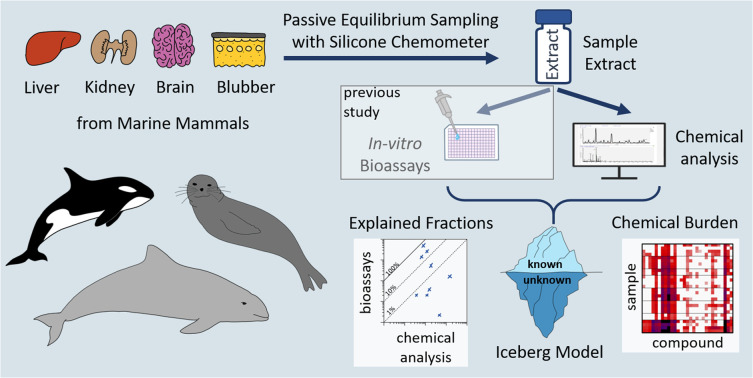

**Environmental significance**

In their natural habitat marine mammals are exposed to a large number and variety of environmental pollutants. Studying the chemical burden of biota tissues and identifying the mixture effects can help in characterizing the internal exposure of chemicals. By using passive equilibrium sampling with silicone with so-called “chemometers” it is possible to transfer environmental mixtures of chemicals into an extract without changing their composition, allowing one to study the mixtures of environmental pollutants extracted from biota and their effects without bias. The combination of *in vitro* bioassays and chemical profiling of legacy and emerging contaminants helps to understand the mixture effects causing the activation of cellular toxicity pathways.

## 1 Introduction

Numerous studies showed that marine mammals are globally exposed to different anthropogenic influences, including encounters with fishery activities (*e.g.* bycatch), noise pollution and exposure to chemicals such as Hydrophobic Organic Compounds (HOCs). HOCs like Polychlorinated Biphenyls (PCBs), Organochlorine Pesticides (OCPs), Polybrominated Diphenyl Ethers (PBDEs), Chlorinated Hydrocarbons (CHCs), and Polycyclic Aromatic Hydrocarbons (PAHs) are known legacy chemicals often found in marine ecosystems.^1–12^ Most HOCs are persistent in the environment, resist biodegradation and can cause adverse effects on aquatic organisms. Due to their natural habitat, long life span, large blubber fraction and elevated trophic level, marine mammals may accumulate a wide variety of HOCs in their tissues, and in some cases they experience adverse health effects.^5,11,13,14^ Already young marine mammals possess a high chemical burden, as lipophilic chemicals are transferred from mother to their offspring during pregnancy and lactation.^11,15–19^ HOCs have a negative impact on the health and survival of marine mammals,^11,20,21^ and can cause, amongst others, immunodeficient effects,^22–25^ reproduction dysfunction,^26–28^ and endocrine disruption.^3,29–31^ The production and use of some HOCs are regulated; they have partly been banned almost two decades ago to prevent adverse environmental and health effects, *e.g.* in the Stockholm Convention on Persistent Organic Pollutants (POPs), including PCBs, hexachlorobenzene (HCB), chemicals from the OCP-group and others.^32^ Besides these regulated legacy POPs, other emerging organic contaminants with similar properties are of concern, especially as many of them are not regulated, *e.g.* personal care products, chemicals with unknown structures, transformation products, and others. Furthermore, mixtures of different chemicals (even at low dose) should be considered as they can jointly elicit effects which can be additive, cumulative or interactive.^33^

To identify and quantify the relevant compounds in tissues of marine mammals, the chemicals need to be extracted from the tissues. A useful tool to transfer environmental mixtures of nonpolar HOCs to different profiling tools, such as chemical analysis and bioanalysis, without changing the chemical composition, is passive equilibrium sampling with a chemometer, *e.g.* the silicone polydimethylsiloxane (PDMS). Chemometers are understood as a common, universal and well-defined polymer reference phase for passive sampling, reaching thermodynamic equilibrium partitioning of a large range of nonpolar organic pollutants in different matrices like biota, sediment and potentially water.^34^ Using chemometers, organic chemicals with a broad range of physicochemical properties are transferred into the extract largely without changing the chemical composition.^35–37^ By using this approach, extracts from biota tissue can conveniently be submitted to instrumental analysis and/or bioanalytical profiling, to analyze the composition or effects of the contained mixture of chemicals. The resulting concentrations in the silicone reference phase can be directly compared across different tissues, individuals and species, circumventing potential bias due to normalization to lipid mass, as would be required with conventional approaches such as traditional exhaustive extraction.^38,39^ Using a chemometer additionally reduces the amount of matrix transferred to the extract and thus a non-destructive cleanup is sufficient to maintain the broad chemical composition in the extract for further analyses.^37,40^

In a previous study,^41^ we examined the chemical mixture effects of silicone chemometers equilibrated with different organs (*i.e.* liver, kidney, brain and blubber) of seven marine mammals from the North and Baltic Seas. Chemometer extracts were tested in three cell-based *in vitro* bioassays to investigate different modes of action: activation of the xenobiotic metabolism, including the activation of (I) the peroxisome proliferator-activated receptor gamma (PPARγ) with the PPARγ-bla GeneBLAzer assay^42^ and (II) the aryl hydrocarbon receptor (AhR) with the AhR-CALUX assay^43^ as well as (III) the adaptive Nrf2-dependent oxidative stress response with the AREc32 assay.^44^ The results indicated that the extracts from liver caused higher bio activation than the corresponding blubber extracts, more precisely 11 ± 0.26 (*n* = 7) times higher activation for PPARγ-bla and 1.9 ± 0.32 (*n* = 4) times higher activation of the AREc32 assay. In the AhR-CALUX the blubber extracts did not activate the AhR up to concentrations where cytotoxicity occurred, whereas for all seven liver extracts an activation for AhR was measured.^41^

The main objective in this study was to submit liver, kidney, brain and blubber tissues from marine mammals to chemometer sampling, cleanup and chemical profiling to determine chemical patterns across different tissues for a broad range of legacy and emerging HOCs. For this purpose, we analyzed the above-mentioned seven marine mammals, sampled the chemicals from organs of five additional marine mammals and compared our results to literature data. Additionally, we assessed the relationship of the mixture effects measured previously with the bioanalytical assays^41^ and the predicted mixture effects of the detected, targeted chemicals of the same sample. To characterize the exposome of the animals, we applied two complementary approaches: broad chemical screening in combination with bioanalytical assessment^41^ of mixture effects. Targeted chemical analysis provides quantitative data regarding a defined number of compounds and can give an impression of the total exposure. By means of bioanalytical testing, mixture effects can be captured and thus the totality of chemicals extracted from a sample can be characterized. Combining both approaches by so-called iceberg modeling allows to estimate which fraction of the observed effect can be explained by the known and quantified chemicals, opposed to which fraction remains unexplained.^45^

## 2 Methods

### 2.1 Biota samples

**Fig. 1:**
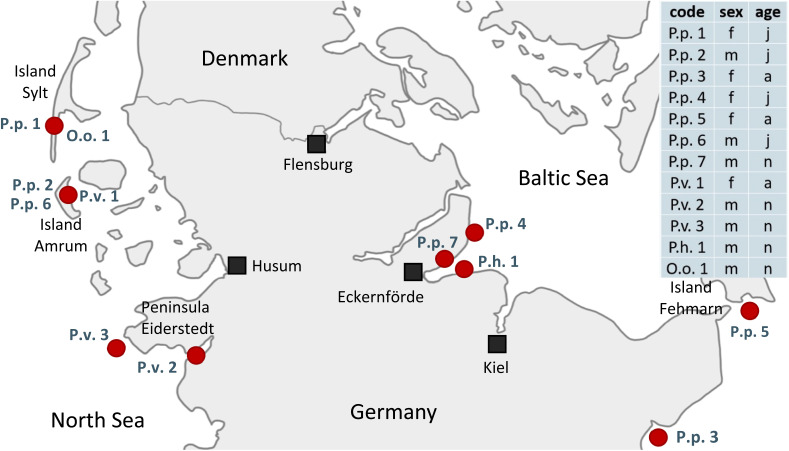
Stranding locations of the twelve examined animals on the shores of Schleswig-Holstein, Germany, and core information of the individuals analyzed: code (P.p.: harbor porpoise, P.v.: harbor seal, P.h.: ringed seal, O.o.: orca, plus running number), sex (f: female, m: male) and age group (a: adult, j: juvenile, n: neonate). For more information, *e.g.* stranding year, available organs and references to previous studies, see Table S1.†

In this study tissues from seven harbor porpoises (*Phocoena phocoena*, P.p.), three harbor seals (*Phoca vitulina*, P.v.), one ringed seal (*Pusa hispida*, P.h.) and one orca (*Orcinus orca*, O.o.) were analyzed. The samples were obtained from deceased animals with different causes of death, *e.g.* stranding, bycatch or pneumonia and final sepsis so that they had to be euthanized, on the German coasts of the North and Baltic Sea between 2016 and 2019. All tissues were collected in moderate to good condition. For eight animals a full set of liver, blubber, brain and kidney, and for four animals a core set of liver and blubber were available. For details on the animals' available organs, sex, age group and stranding location, see Fig. 1 and Table S1.† Samples were abbreviated with their species name (P.p., P.v., P.h. and O.o.) plus a running number. For coherent sample labeling and to facilitate direct comparison, samples analyzed in Reiter *et al.*^41^ were termed with the identical name (P.p.1–5, P.v.1, O.o.1) as before. The additional samples received a running number. The order of the samples was random. The tissues were processed as described in detail by Reiter *et al.*^41^ Briefly, the samples were homogenized with a blender (see Table S2†) and stored at −20 °C. For all samples the lipid content was determined gravimetrically following extraction with various mixtures of solvents;^46^ for the detailed method, see Text S1.†

### 2.2 Solvents and standards

If not otherwise specified, ethyl acetate (EtAc), acetonitrile, *n*-hexane and isopropanol of gas chromatography grade were purchased from Merck, Darmstadt, Germany and used. Diethyl ether (PESTINORM®) was purchased from VWR International, Darmstadt, Germany. Analytical standards of at least 98% purity were purchased from Dr Ehrenstorfer (Augsburg, Germany) and from Sigma-Aldrich (Germany), stable isotope-labeled standards were purchased from Wellington Laboratories (Guelph, ON, Canada) and Campro Scientific (Berlin, Germany). All standards are listed in Table S3.†

### 2.3 Extraction of chemicals

#### 2.3.1 Chemometer sampling

Chemometer sampling and extraction was performed with the silicone PDMS according to previously published studies.^41,47,48^ Briefly, PDMS sheets (SSP-M823, Special Silicone Products, Ballston, USA) with thicknesses of 125 μm, 250 μm and 350 μm were cut (see Table S2† for dimensions), solvent-cleaned using Soxhlet extraction with EtAc (Honeywell, HPLC grade) for at least 20 hours and stored in fresh EtAc (Honeywell, HPLC grade) at room temperature until usage. Before chemometer sampling, the sheets were air-dried under the fume hood for approximately 2 h and the PDMS weight was determined with a micro-analytical balance (Mettler Toledo, Gießen, Germany). The equilibration of the chemometers with the oil-like blubber homogenate was performed statically. The PDMS was immersed in a vial with homogenized blubber and equilibrated for 48 h at 4 °C; except for the blubber tissue from O.o.1 that was equilibrated for 72 h, due to lower lipid content (Table S2†), to make sure equilibrium partitioning between the sample and the chemometer was approached.^41^ To equilibrate the PDMS with tissue from liver, brain and kidney, dynamic sampling with manual relocations was carried out.^41,48^ The PDMS sheets were layered between the homogenized tissue, regularly relocated and the tissue mixed, *i.e.* eight to ten relocations per day (every 90 to 110 minutes) were performed and samples left static overnight. During the experiment, the samples were kept at 4 °C for seven to nine days. Up to 70 relocations were carried out unless the texture of the tissue homogenate did not allow any more for continued sampling. The maximum mass of PDMS to be used in each single sample met the negligible depletion conditions (<5% depletion of pollutants by partitioning to PDMS).^49^ For selected tissues of sufficient mass, we sampled duplicates of 125 μm, 250 μm and 350 μm thick PDMS sheets each; for the other tissues, duplicates of 350 μm sheets were taken. See Table S2† for detailed PDMS sheet replicate numbers, dimensions and weights, as well as the number of relocations and the total exposure time.

After equilibrating the chemometers with the tissue, the sheets were removed from the tissue and their surface was thoroughly cleaned with lint-free tissues. The PDMS sheets were briefly cleaned with bidistilled water twice and dried with lint-free tissues. The weights of the PDMS sheets were recorded to individually document lipid uptake into the PDMS. For extraction, 1 mL EtAc per 100 mg of PDMS was used; or a minimum volume of 1.5 mL of EtAc for the thinner PDMS sheets to ensure that the sheet was fully immersed in the solvent. Solvent extraction was performed twice for at least 2 h on a horizontal roller mixer and each extract was collected and stored at 4 °C for further processing.

To reduce the amount of co-extracted lipids in the extracts (due to lipid uptake into the PDMS), the samples were submitted to a non-destructive cleanup.^40^ As described before,^41^ due to matrix interferences in either the bioanalytical test system or the instrumental analysis, different cleanup procedures were required. For bioanalytical measurements a freeze-out cleanup^40^ in combination with a primary secondary amine (PSA) sorbent (Agilent Technologies, USA) extraction,^50^ was used; for chemical analysis, a combination of Captiva EMR-Lipid cartridges (3 mL, Agilent Technologies, USA)^40^ and PSA extraction was preferred. The cleanup procedures are described in detail in Text S2.† In an ideal case, the extracts dosed to the bioassays would have been treated in the identical way as the extracts for chemical analysis. Due to cytotoxic effects that occurred after the EMR cleanup, freeze-out cleanup needed to be applied for the bioanalytical measurements.^41^ The other way around, for instrumental GC-HRMS measurement, an EMR cleanup was necessary to avoid interferences with the instrument performance and lifetime. In spite of different applied cleanup procedures, the recoveries of the analyzed chemicals between EMR and freeze-out were similar, as described in Muz *et al.*^40^ and Text S2.†

After the cleanup the extracts were blown down to dryness and the residue dissolved in EtAc, spiked with a mixture of 21 isotope-labeled internal standards (50–100 ng mL^−1^, see Table S3†) and stored at −20 °C until analysis.

#### 2.3.2 Exhaustive solvent extraction

In addition to passive equilibrium sampling, homogenized blubber tissues of the animals P.p.1–5, P.v.1 and O.o.1 were solvent-extracted with the “modified II method” described by Jensen *et al.*^51^ Briefly, approximately 10 mg of blubber tissue was extracted in three processing steps with different mixtures of 2-propanol, diethyl ether and *n*-hexane. The collected extract was dried and weighed to determine the extracted lipid weight (micro-analytical balance). As for the chemometers extracts, the extracts were submitted to a non-destructive cleanup (EMR-Lipid cartridges and PSA extraction, see Text S2†) and were finally spiked with isotope-labeled internal standards (100 ng mL^−1^, Table S3†) and stored at −20 °C until analysis.

### 2.4 Chemical analysis

All samples were analyzed by gas chromatography-high resolution mass spectrometry (GC-HRMS, QExactive, Thermo Fisher Scientific, Germany), as described elsewhere.^41,52^ In total, 117 target compounds, covering a broad range of physicochemical properties and targeting different substance classes of both legacy and emerging hydrophobic organic pollutants, were investigated in our study. The chemical analysis covered legacy and emerging HOCs such as PCBs, PAHs, OCPs, BDEs, Pyrethroids, CHCs, Musks, and other compounds, including antioxidants, UV filters and long-chain chemicals, categorized as “Others” (listed in Table S3†). Detailed instrumental conditions are provided in Text S3.† The target list addressed the compounds found to be relevant in an earlier environmental study using chemometers, analyzing these contaminants in marine sediment^52^ as well as in other studies currently ongoing in fresh water biota. Therefore, the detected chemicals in these studies are likely to occur in marine mammals.

Method Detection Limits (MDLs, Table S4†), were determined using a two-tailed *t*-distribution test with 99% intervals, based on the US EPA guidelines, described in detail in Text S4.†^53^ Extract concentration of target compounds below the MDL were considered as not detected (n.d.) for further data evaluation. Furthermore, blank subtraction and correction for lipid uptake into the PDMS sheets were carried out. Details on the data evaluation are described in Text S4.† Quality assurance/quality control procedures were in place for the GC-HRMS instrument and the analysis method, including standard operating procedures, trained technicians dedicated to the equipment and traceability (incl. analytical standards and reference materials whenever available), and are described in detail in Text S4.†

#### 2.4.1 Data evaluation: conversion of silicone-based concentrations *c*_PDMS_ to lipid-based concentrations at equilibrium with the tissue *c*_lipid, eq._

In this study, the concentration are reported as silicone-based concentrations *c*_PDMS_ in mass_analyte_ per mass_PDMS._ The PDMS chemometers were used as a common reference phase, circumventing potential bias due to normalization to lipid mass, given that concentrations between the equilibrated chemometers are directly compared. However, the traditional way to report chemical burden in biota is *via* lipid-based concentrations *c*_lipid_ in mass_analyte_ per mass_lipid_. Thus, in order to compare the concentrations found in this study with those from literature and to make the results accessible for other researchers, the *c*_PDMS_ was converted to *c*_lipid, eq._ using compound-specific lipid/PDMS partition coefficients *K*_lipid/PDMS_ (eqn (1)). For conversion, experimentally determined *K*_lipid/PDMS_ values are available for 31 compounds (13 PCBs, 8 PAHs, 8 OCPs and 2 CHCs).^36,54^ To translate the concentrations for those compounds that were without an experimentally determined partition coefficient, a common method is to use an average value.^52,55,56^ For this study, we calculated the average from the experimentally determined *K*_lipid/PDMS_ values from Smedes *et al.*,^54^ for the compounds that were detected in this study (excluding the HCH isomers),^57^ resulting in a mean *K*_lipid/PDMS_ of 23 (*n* = 25). This approximation to an average value agrees with using the theoretical average *K*_lipid/PDMS_ for all the compounds using modeled values from the UFZ-LSER database^58^ (Table S4†). To prove that the converted values using the partition coefficients were appropriate, we compared the converted *c*_lipid, eq._ values, using experimentally determined *K*_lipid/PDMS_, with our measured *c*_lipid_ values from total exhaustive extraction (Table S6†). The results are described in detail in Text S5.†

1 *c*_lipid, eq._ = *K*_lipid/PDMS_ × *c*_PDMS_^−1^

### 2.5 Iceberg modeling

In our previous study,^41^ PDMS extracts from the organs of P.p.1–5, P.v.1 and O.o.1 were measured in three cell-based *in vitro* bioassays: the PPARγ-bla GeneBLAzer assay,^42^ AhR-CALUX assay^43^ and AREc32 assay.^44^ With the earlier generated data^41^ and the data from the chemical analysis, iceberg modeling was carried out. The detailed description of the iceberg modeling is given in Text S6.† Briefly, the bioanalytical equivalent concentrations (BEQ) measured from the results of the cell-based *in vitro* bioassays (BEQ_bio_, eqn S4†), reported in our earlier study,^41^ were compared with the predicted effect, calculated by the sum of detected compounds in the extract (BEQ_chem_, eqn S5†). The ratio of BEQ_chem_/BEQ_bio_ indicates which fraction of the measured effect in the bioassay can be explained by the detected compounds. In addition, iceberg modeling was applied to the cytotoxic effects, detected in the AhR-CALUX, by the ratio of the predicted Toxic Unit (TU) of all detected compounds, TU_chem_, and the bioanalytically measured TU_bio_ in the AhR-CALUX (TU_chem_/TU_bio_, eqn S7†).

### 2.6 Statistical evaluations

To calculate differences between variables of two different organs, a ratio paired *t*-test was used. Gaussian normal distribution of the results was confirmed. Statistical significance was defined with a *p*-value <0.05 (*). The *t*-test was calculated with the Software GraphPad Prism V 9.5.0.

## 3 Results and discussion

### 3.1 Characterization of tissue and lipid weight gain

The lipid content varied greatly between and within tissues, *i.e.* 37 to 101 g_lipid_ g_blubber_^−1^, 1.9 to 23 g_lipid_ g_liver_^−1^, 6.8 to 12 g_lipid_ g_brain_^−1^, 2.0 to 3.9 g_lipid_ g_kidney_^−1^ (Table S2†). *In tissue* passive sampling comes along with a weight gain of the PDMS due to lipid uptake into the polymer.^36^ Although the lipid content of the investigated tissues ranged from 1.9 to 101%, the lipid uptake into the PDMS was rather uniform, *i.e.* 0.88 ± 0.62% (mean ± SD, *n* = 40). The lipid uptake into the PDMS thus was independent of the lipid content of the tissue but tissue-specific, *e.g.* lower lipid uptake was observed for brain tissue (0.30 ± 0.26% (*n* = 8)) than for kidney tissue (0.64 ± 0.24% (*n* = 8)), although the lipid content of the brain was higher than that of the kidney (Fig. S1†).

### 3.2 Chemical burden of marine mammals

**Fig. 2:**
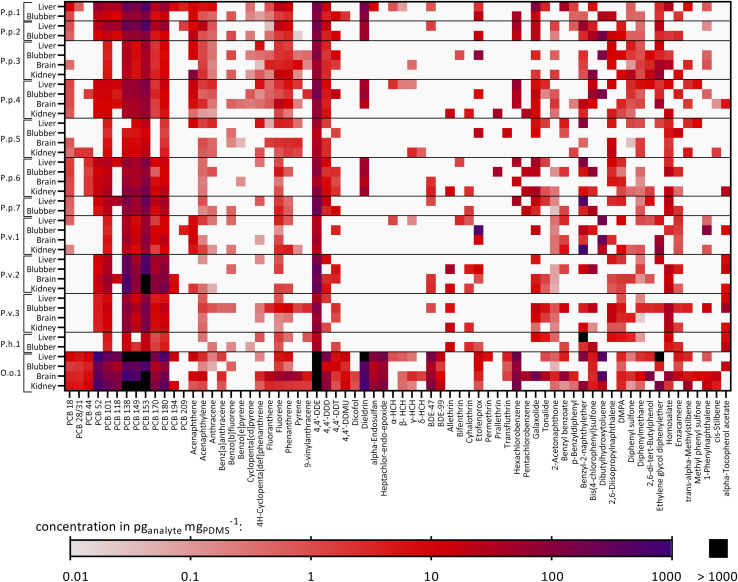
Heatmap showing the detected targeted compounds found in each animal and their corresponding organs: P.p.: harbor porpoise, P.v.: harbor seal, P.h.: ringed seal, O.o.: orca, plus running number. White: the chemical was not found in the sample. Concentrations in pg_analyte_ mg_PDMS_^−1^. Concentrations above 1000 pg_analyte_ mg_PDMS_^−1^ are colored in black. Furthermore, some extracts exceeded the maximal concentration of the highest concentrated calibration solutions and hence, have a semi-quantitative character (discussed in detail in Text S6†). Affected samples for which every replicate exceeded the calibration are P.p.1 liver: PCB153; P.p.7 liver: benzyl-2-naphthylether; P.v.2 all organs: PCB153; P.v.2 brain, kidney: PCB138; P.h.1 liver: benzyl-2-naphthylether; O.o.1 liver: PCB52, dieldrin, ethylene glycol diphenylether; O.o.1 liver, brain, kidney: PCBs 138, 149, 153; O.o.1 all organs: 4,4′-DDE. Detailed data are given in Table S5.†

The chemical concentrations in the PDMS sheets, *c*_PDMS_ in pg_analyte_ mg_PDMS_^−1^, were calculated and are summarized in Table S5.† 70 out of 117 target compounds were detected in at least one out of 40 samples and thus 47 targeted chemicals were not found in any sample. Fig. 2 shows a heatmap with the detected targeted chemicals and the samples in which they were found. Additionally, the total chemical burden of the different compound groups is shown in Fig. S2.† Eight compounds were found in 90% or more of the samples (*i.e.*, 36–40 samples), six of which were PCBs (PCB 101, 138, 149, 153, 170, 180), one PAH (fluorene) and one OCP (4,4′-DDE). Compounds that were found in particularly high concentrations in most samples were PCB 153 (geometric mean ± geometric SD: 157 ± 3.98 pg mg_PDMS_^−1^, *n* = 40), PCB 138 (90.2 ± 3.99 pg mg_PDMS_^−1^, *n* = 39), 4,4′-DDE (142 ± 5.16 pg mg_PDMS_^−1^, *n* = 40) and dieldrin (115 ± 3.64 pg mg_PDMS_^−1^, *n* = 15). The total burden of the samples varied greatly between animals and organs. Exceptionally high concentrations were found in O.o.1, a neonate orca stranded and found in the North Sea (especially PCBs, DDX (4,4′-DDE, 4,4′-DDD and 4,4′-DDT) and BDEs), see Fig. 2. High concentrations of PCBs in this individuum were also observed in an earlier study by Schnitzler *et al.*^59^ The lowest chemical burden was found in the two adult female harbor porpoises, stranded and found in the Baltic Sea, P.p.3 and P.p.5, and in the neonate male ringed seal P.h.1 from the Baltic Sea. The concentrations of selected chemicals found in some extracts were above the maximal concentration of the calibration which is discussed in detail in Text S7.† Consequently, the extract concentrations of these compounds were extrapolated from the linear concentration curve; the single extracts that exceeded the maximal calibration of a compound are highlighted in Table S5.†

Comparing the tissues within each animal revealed that some of the compounds tended to accumulate in the four organs to different extents. The compound 4,4′-DDT was found in every blubber sample from all analyzed individuals (*n* = 12) and in five (out of eight) brain samples, whereas it was only detected in two (out of eight) kidney samples and one liver sample (out of twelve). As blubber and brain are less metabolically active than liver and kidney, this was an expected observation. Another compound that was frequently found in blubber samples was the UV filter enzacamene (4-methylbenzylidene camphor, *n* = 11), which was also found in five kidney samples, whereas it was rarely found in liver and brain (each *n* = 2). Enzacamene is used in personal care products and was detected before in dolphins from the Brazilian coast.^60,61^ Furthermore, the pyrethroid etofenprox (often used as insecticide) and the PAH benzo[*b*]fluorene were frequently found in blubber (*n* = 9), whereas both compounds were less frequently detected in the other organs (*n* = 1–3). Etofenprox and pyrethroids in general can be metabolized by mammals, explaining the higher detection frequency in the less metabolically active blubber tissue.^62^

The other way around, the polycyclic musk tonalide was found in more than half of the liver samples (*n* = 7) but it was detected less frequently in the other organs (*n* = 2–3). Hexachlorocyclohexanes (HCHs, α-, β-, γ- and δ-isomers), were not found in blubber, whereas the α-isomer was found in four liver samples, the β-isomer in three liver samples, one kidney and one brain samples, the γ-isomer in three liver samples, two kidney and brain samples and the δ-isomer in one kidney and brain sample. Still, HCHs were detected in earlier studies in blubber samples from harbor porpoises from the Baltic and North Seas.^63,64^

Fig. S3† illustrates the specific contaminant patterns of each organ and animal. Overall, a high fraction of PCBs (mean ± SD: 44 ± 22%), OCPs (24 ± 16%) and chemicals from the “Others” category (incl. antioxidants, UV filters and other compounds) (22 ± 22%) was detected in all samples. This pattern was similar for all organs, except for blubber samples, where a higher pyrethroid fraction was additionally identified (12 ± 19%); contrarily, pyrethroids were found in less than 1% of the liver and brain samples and on average in 4.9 ± 7.2% in the kidney samples.

### 3.3 Chemical distribution between liver and blubber

Paired liver and blubber samples were available for all twelve animals. In our previous work,^41^ the bioanalytical results of paired liver and blubber samples from P.p.1–5, P.v.1 and O.o.1 indicated a higher activation of the cell assays from liver extracts, compared to the corresponding blubber samples; more specifically, for PPARγ-bla by a factor of 11 ± 0.26 (*n* = 7), for AREc32 by a factor of 1.9 ± 0.32 (*n* = 4) and in the AhR-CALUX activation was only measured for liver extracts (*n* = 7), whereas the blubber extracts did not activate the AhR up to concentrations where cytotoxicity occurred. In this work, a ratio paired *t*-test was used to compare the concentrations, measured with GC-HRMS, in the chemometers equilibrated with the liver (*c*_PDMS, liver_), to the corresponding equilibrated blubber sample (*c*_PDMS, blubber_), describing the liver/blubber chemical activity ratio, *c*_PDMS, liver_/*c*_PDMS, blubber_. For ∑PCB_13_, a significantly (*, *p* < 0.05) higher concentration of 1.6 times (95% CI: 1.0 to 2.5, *, *n* = 12) was found in *c*_PDMS, liver_, compared to the corresponding *c*_PDMS, blubber_, *e.g.* PCB153 as the PCB congener found in the highest concentration in all samples, was quantified at 1.8 times (1.1 to 2.8, *, *n* = 12) higher concentrations compared to *c*_PDMS, blubber_. Also, for HCB, which was only found in three pairs of liver and blubber, a liver/blubber chemical activity ratio of 2.3 (1.5 to 3.6, *, *n* = 3) was determined. As for the PAHs, *c*_PDMS, liver_ was on average 1.6 (0.58 to 5.2, *n* = 12) times higher, *e.g.* for fluorene 1.7 times (0.50 to 5.9, *n* = 11) higher, compared to *c*_PDMS, blubber_. Regarding the musk compounds, the liver/blubber chemical activity ratio was 1.8 (0.57 to 5.5, *n* = 6), *e.g.*, for galaxolide 1.6 (0.5 to 5.2, *n* = 6). The other way around considering the blubber/liver chemical activity ratio (*c*_PDMS, blubber_/*c*_PDMS, liver_), for pyrethroids a 13 times (0.25 to 666, *n* = 4) higher *c*_PDMS, blubber_ was found compared to the corresponding *c*_PDMS, liver_. Liver/blubber chemical activity ratios close to unity were found for OCPs (0.95 (0.58 to 1.5, *n* = 12)). The importance to measure the chemical burden in different organs of animals is emphasized by the fact that different patterns are observed in different organs.

Liver/blubber chemical activity ratios are shown for selected compounds and for all animals in Fig. S4.† Compounds like the UV filter homosalate, enzacamene, 4,4′-DDT, the insecticide etofenprox and alpha-tocopherol acetate, a synthetic form of vitamin E, were found in higher concentrations in blubber than in liver. Contrarily, compounds like HCB, 4,4′-DDD, PCB52 and PCB101 tended to be found at higher concentrations in liver. Overall, most compounds showed no clear tendency to accumulate in any organ. Furthermore, there seemed not to be any systematic differences between the organs or a correlation to the chemicals' octanol/water partition coefficients, *K*_ow_ (see Fig. S4†).

Most compounds were close to equilibrium partitioning within the organism (chemical activity ratio = 1), indicating that the tissues within the organism are at equilibrium. For O.o.1 most compounds were found in higher concentration in liver than in blubber (Fig. S4†), indicating a more recent exposure of the bioaccumulative HOCs, as partitioning to blubber was not yet in equilibrium.^65^ This individuum was only a few days old when it stranded,^66^ which could be the reason for consistently lower concentrations of HOCs in blubber.

### 3.4 Integration of chemical data to previously measured data from literature

The lipid-normalized sum concentrations for all 13 PCBs (∑PCB_13_) in blubber tissues ranged from 0.64 mg kg_lipid_^−1^ in P.p.5 to 44.5 mg kg_lipid_^−1^ in O.o.1. For all individuals, a geometric mean (± geometric SD) of 5.85 ± 3.36 mg kg_lipid_^−1^ was determined. The concentrations found in the present study correspond well with results from previous studies, see Table 1. In contrast, Schnitzler *et al.*^59^ reported 225 mg kg_lipid_^−1^ (∑PCB_28_) or 176 mg kg_lipid_^−1^ (∑PCB_12_, matching with the PCBs in this study, without PCB 18) for the neonate orca also analyzed here (O.o.1). When comparing the *c*_lipid_ from the exhaustive solvent extraction in this study (Table S6†), a ∑PCB_12_ of 63.3 mg kg_lipid_^−1^ was determined, which is 3 times lower than the values reported earlier. Interestingly, lipid contents of the blubber samples analyzed by Schnitzler *et al.* (16–22%, *n* = 3) were approximately 2 times lower than of the blubber samples analyzed in this study (35–39%, *n* = 2), which could indicate that necropsy samples from different layers of the blubber were studied, providing an explanation for (part of) the discrepancy. According to Sørmo *et al.*,^17^ the concentration of pollutants in the different layers of blubber tissue from marine mammals can differ, due to distinct lipid compositions and metabolic activities. Although the full blubber layer between skin and muscle fascia was sampled during the necropsy, the distributed subsamples could still differ in composition (as is indicated in the different lipid contents) that might yield in mismatched results.

**Table 1:** Data from selected published studies on the chemical burden in harbor porpoise blubber. Concentrations are reported as normalized to the lipid weight (mg kg_lipid_^−1^) and in a range from minimum to maximum concentrations. Sample details imply numbers of individuals (*n*), sampling location, years and the age and sex of the animals. ΣPCB_*x*_: sum concentrations for *x* PCB congeners, ΣDDX_6_: sum concentrations for 2,4′-DDD, 2,4′-DDT, 2,4′-DDE, 4,4′-DDD, 4,4′-DDE, 4,4′-DDT

Concentration range of the compound (group) reported in mg kg_lipid_^−1^	Sample details	Reference
**Polychlorinated biphenyls (PCBs)**		
∑PCB_17_: 0.21–90	*n* = 112, Danish waters, 2003–2019, different age groups, male and female	11
∑PCB_35_: 1.1–82	*n* = 28, German North Sea, 1990–2008, different age groups, male and female	69

**Organochlorine pesticides (OCPs)**		
∑DDX_6_: 0.4–22.9	*n* = 28, German North Sea, 1990–2008, different age groups, male and female	69
4,4′-DDE: 0.59–12	*n* = 29, North and Baltic Sea, 1994–1995, mostly immature animals, male and female	63

**Hexachlorobenzene (HCB)**		
HCB: 0.10–0.92	*n* = 29, North and Baltic Sea, 1994–1995, mostly immature animals, male and female	63
HCB: 0.013–0.42	*n* = 34, Danish waters, 2003–2019, different age groups, male and female	11

DDXs often mark the largest share of the OCP-burden,^63,67,68^ and the lipid-normalized sum concentrations of 4,4′-DDE, 4,4′-DDD and 4,4′-DDT (∑DDX_3_) in blubber tissues in this study ranged from 0.45 mg kg_lipid_^−1^ in P.p.5 to 6.3 mg kg_lipid_^−1^ in P.v.2. Only O.o.1 exceeded this range by a factor of 11 at 74.4 mg kg_lipid_^−1^. Among the DDXs, 4,4′-DDE was the most abundant representative, which is a common pattern in marine mammals,^67,68^ and ranged from 0.40 to 4.3 mg kg_lipid_^−1^. Except for O.o.1, the DDX concentrations determined in this study correspond with previously reported results, listed in Table 1.

HCB was only found in five out of the seven harbor porpoise samples as well as in the orca, but not in the harbor seal or ringed seal samples. Concentrations in the respective blubber tissues ranged from 0.22 to 0.88 mg kg_lipid_^−1^. This range is consistent with the scale of observations made in several older and recent studies, summarized in Table 1.

It is important to put the different concentrations into context with known thresholds of physiological impacts in marine mammals, which are based on mixture effects. At present, there are three commonly used ∑PCB thresholds that provide guidance for assessing PCB contamination of marine mammal tissues: (I) 9.0 mg kg_lipid_^−1^ (∑PCB_23_), which marks the general onset of physiological impacts in marine mammals,^70,71^ (II) 11.0 mg kg_lipid_^−1^ (∑PCB_25_), from which on infertility and reproductive failure can be expected in female sexually mature harbor porpoises^11,72^ and (III) 41.0 mg kg_lipid_^−1^ (∑PCB_23_), for the onset of profound reproductive impairment of Baltic ringed seals.^71,73^

The lowest threshold (I) was transgressed by 14 samples and in at least one organ from P.p.1, P.p.2, P.p.3, P.p.6, P.p.7, P.v.2, O.o.1. The second lowest threshold (II) was transgressed by 12 samples by the same individuals mentioned above. The highest threshold (III) was transgressed by all examined organs from O.o.1 and in the brain and kidney of P.v.2. It is important to notice that in this study, the ∑PCB trespassing some thresholds is corresponding to ∑PCB_13_ and not to ∑PCB_23_ or ∑PCB_25_, respectively. A summary is shown in Fig. S6.† In the past decades, numerous studies have found PCB levels in marine mammals that are believed to trigger various adverse effects.^3,29,74,75^ For instance, Das *et al.*^3^ hypothesized that concentrations as low as 7.66 ± 5.08 mg kg_lipid_^−1^ (∑PCB_6_) interfere with the harbor porpoise thyroid functions leading to severe interfollicular fibrosis. This threshold was transgressed in at least one organ of eight animals, namely P.p.1, P.p.2, P.p.4, P.p.6, P.p.7, P.v.2, P.v.3 and O.o.1. Recently, it has been specified that the risk of death from infectious diseases is raised by 5% for every 1 mg kg_lipid_^−1^ increase of the ∑PCB_25_ burden, which poses risk to all marine mammals.^75^

As PAHs are metabolized in higher organisms, elevated concentrations of PAHs might primarily reflect on recent exposure.^76^ In this study the contamination levels of PAHs relative to the total chemical burden in blubber were between 0.041–4.2% and hence might not indicate a major recent exposure. However, carcinogenic properties of PAHs have been identified in mammals, but to the best of our knowledge, no effect thresholds have been established for marine mammals.^67,77^

Limited thresholds for adverse effects on marine mammals have been established for OCPs, however, several studies indicated that OCPs adversely affect marine mammals, *e.g.* by impairing thyroid function or by acting as endocrine disrupting chemicals potentially affecting the reproduction.^3,68^ Furthermore, 4,4′-DDT levels of 2.44 ± 0.37 mg kg_lipid_^−1^ found in Baltic harbor seals are suspected to trigger immunotoxic effects,^78^ but no exceedance was observed in the blubber samples analyzed in this study. Furthermore, organohalogen compounds, such as DDX, are considered toxic from concentrations of 1 mg kg_wet weight_^−1^.^79^ Considering the lipid content of each blubber sample (Table S1†), nine (out of twelve) blubber samples (P.p.1, P.p.2, P.p.3, P.p.4, P.p.6, P.p.7, P.v.2, P.p.3, O.o.1) exceeded this threshold for ∑DDX_3_, suggesting that 75% of the analyzed individuals could have suffered from adverse effects caused by their ∑DDX_3_ burden.

### 3.5 Iceberg modeling

To determine how much of the bioanalytically measured mixture effect (BEQ_bio_)^41^ can be explained by analytically determined chemicals and their predicted mixture effect (BEQ_chem_), iceberg modeling was applied. In addition, for the AhR-CALUX assay, cytotoxic effects from bioanalysis (TU_bio_) and the predicted cytotoxic effects from chemical analysis (TU_chem_) were compared. The bioanalytical results for PPARγ-bla, AhR and AREc32 were reported and discussed in detail in an earlier study.^41^ Values used for the iceberg modeling are summarized in Table S8.†

From the 70 detected compounds, 9 chemicals cannot be captured in bioassays because they are too volatile (*K*_medium/air_ below a threshold of 10^4^, Fig. S7 and Table S4†).^80^ From the 59 remaining chemicals, effect data for 37 compounds were available (Table S4†), of which 11 chemicals did not activate one of the three bioassays, and no cytotoxic effects were measurable for the AhR-CALUX. A fraction between 27–95% (geometric mean ± geometric SD: 77 ± 1.3%), normalized to the total concentration, could be captured with the 37 compounds with known effect data. Consequently between 2.2–72% (11 ± 2.3%) remained unknown, due to unavailable compounds (24 chemicals) and between 0.58–28% (3.9 ± 2.7%) stayed unexplained due to chemicals with explicit volatility, as shown in Fig. S8.† For most extracts the major compound burden could be captured with the effect data, although especially for the extracts of P.p.5 liver and P.v.1 kidney, more than 50% could not be explained.

**Fig. 3:**
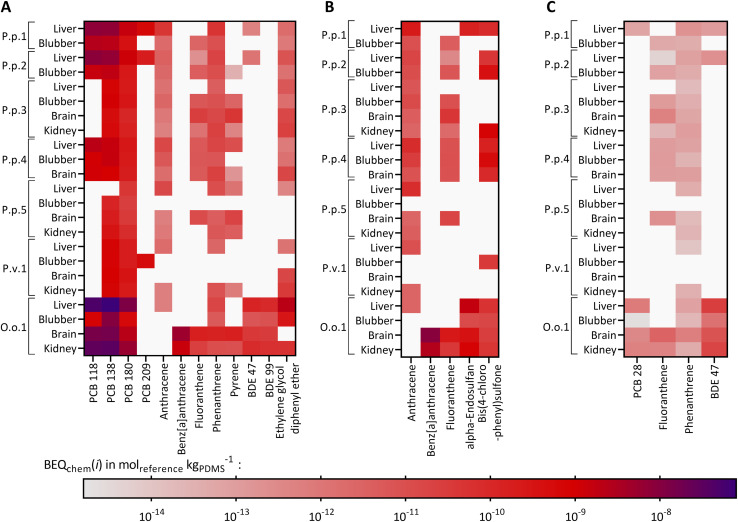
Heatmap of compounds activating the (A) AhR-CALUX bioassay, (B) oxidative stress response in the AREc32 bioassay and (C) the PPARγ-bla bioassay, as BEQ_chem_(*i*) of the compound's activation of the assay in mol_reference_ kg_PDMS_^−1^ for the analyzed animals and their corresponding organs: P.p.: harbor porpoise, P.v.: harbor seal, O.o.: orca, plus running number. White: the chemical was not found in the sample. Reference chemicals for AhR-CALUX and AREc32 are benzo[*a*]pyrene and for PPARγ-bla Rosiglitazone.

**Fig. 4:**
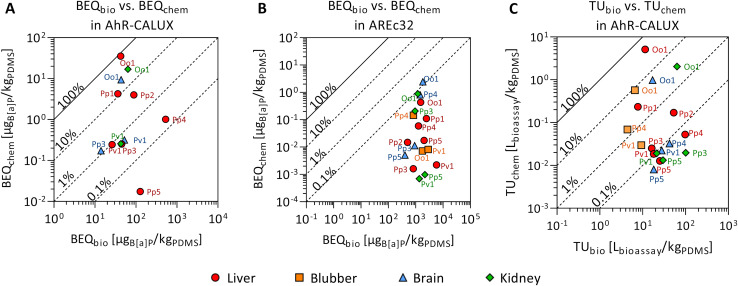
Mixture effects from liver (red circle), blubber (orange square), brain (blue triangle) and kidney (green diamond) samples from seven marine mammals (P.p.: harbor porpoise, P.v.: harbor seal, P.h.: ringed seal, O.o.: orca, plus running number). Comparison of the predicted mixture effect from the detected compounds, described as bioanalytical equivalent concentration, BEQ_chem_, to the bioanalytically derived mixture effects, BEQ_bio_, in the (A) AhR-CALUX and (B) AREc32 assays. Reference chemical for AhR-CALUX and AREc32 is benzo[*a*]pyrene (B[*a*]P). (C) Comparison of the cytotoxic effects measured in the AhR-CALUX assay (TU_bio_) with the predicted effect (TU_chem_), data in Tables S4 and S8.† The symbols are labeled with the corresponding code from the different animals.

Twelve compounds of the detected chemicals are known to activate the AhR-CALUX, *i.e.*, 4 PCBs, 5 PAHs, 2 BDEs, and ethylene glycol diphenyl ether. The PCB congeners 118, 138 and 180 explained most of the effect, whereas the other compounds in the extracts played a minor role (Fig. 3A). Overall, BEQ_chem_ explained between 0.014–83% of the AhR-activating effect (BEQ_bio_) by the 12 compounds (Fig. 4A). Most of the effect was explained for the liver extract of O.o.1 (83%), but also for the corresponding brain and kidney samples, a substantial fraction was explained (22% and 26%). Between 1.2 and 12% of the effect was explained for the liver extracts of P.p.1 and P.p.2 and the brain extract of P.p.3. Less than 1% AhR-activation was determined in liver extracts of P.p.3, P.p.4, P.p.5 and all extracts of P.v.1. The smallest fraction was explained in the liver extract of P.p.5 with 0.014%. This observation correlates with the overall chemical burden, as the highest compound concentrations were found in O.o.1, whereas low concentrations were detected in the extracts from P.p.5. Chemicals that are known to activate the AhR to a great extent are dioxin-like compounds like polychlorinated dibenzodioxins and dibenzofurans, which were not targeted in this study. However, these compounds were detected in marine mammals from the Baltic and North Sea before and thus could play a role in the measured activation of the AhR.^63,81^

For iceberg modeling of the induction of oxidative stress, measured with the AREc32 assay, effect data from five compounds were available: anthracene, benz[*a*]anthracene, fluoranthene, alpha-endosulfan and bis(4-chlorophenyl)sulfone (Fig. 3B). As none of these compounds were detected in the extracts of P.p.5 blubber and P.v.1 brain, no fraction of the observed effect could be explained for these samples. Less than 0.13% was explained for all other samples (Fig. 4B). The Nrf2-dependent oxidative stress response can be activated through various pathways and thus many different chemicals may activate the AREc32 assay. Environmental pollutants such as some PAHs or quinones are known to activate the oxidative stress response pathway, and, as for the AhR, polychlorinated dibenzodioxins.^44,82^

PPARγ is typically activated by long-chain carboxylic acids such as perfluorinated alkane acids and endogenous lipids.^83^ Therefore, iceberg modeling is not meaningful for the detected chemicals, only few of which were active in PPARg (PCB28, fluoranthene, phenanthrene and BDE47, shown in Fig. 3C) and consequently negligible fractions (<0.1%) of PPARγ-activation could be explained by the detected chemicals (Fig. S9†). Former studies showed that marine mammals from German Seas were contaminated with polyfluorinated compounds, which activate the PPARγ and could explain part of the observed effect;^8,84,85^ however, these compounds were not targeted in this study.

**Fig. 5:**
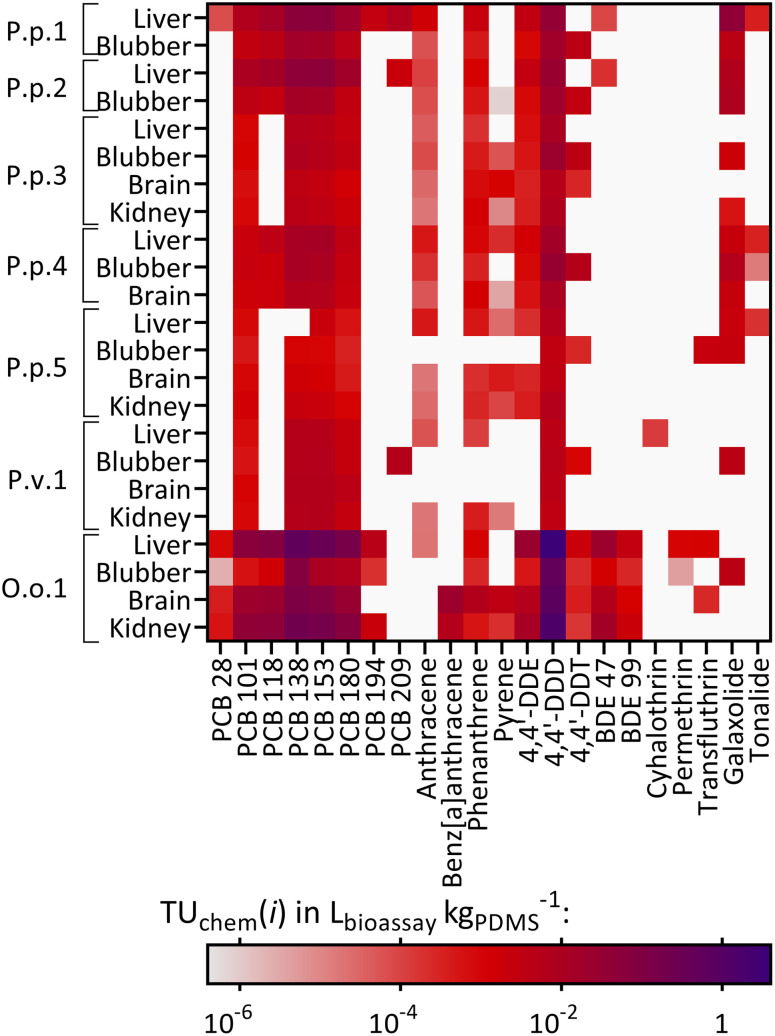
Heatmap of the compounds causing cytotoxicity in the AhR-CALUX bioassay in chemometers equilibrated with the animals and their corresponding organs: P.p.: harbor porpoise, P.v.: harbor seal, O.o.: orca, plus running number, described as TU_chem_(*i*) in L_bioassay_ kg_PDMS_^−1^. White: the chemical was not found in the sample.

Cytotoxic effects (TU_bio_) were mainly measured in the AhR-CALUX assay. Amongst the detected chemicals, 22 were characterized to induce cytotoxic effects for the AhR-CALUX cell line; more specifically: 8 PCBs, 4 PAHs, 3 DDXs, 2 BDEs, 3 pyrethroids and 2 musks. The compounds dominating the TU_chem_ were PCB congeners 101, 118, 138, 135, 180 and 4,4′-DDD (Fig. 5). As a result, between 0.044–45% of the cytotoxic effects were explained when comparing TU_chem_ and the measured TU_bio_ (Fig. 4C). Similar to the specific mode of action, a larger fraction of the effect was explained for the tissue samples of O.o.1 with the high chemical burden (3.2–45%), whereas the smallest fractions were explained for the tissue extracts of the low contaminated P.p.5 (0.044–0.050%).

The animal with the highest chemical burden in this study was the orca O.o.1 and thus a larger fraction of the observed effects was explained for the tissues of this animal than for the remaining individuals. Contrarily, for P.p.3 and P.p.5, as the animals with the lowest overall concentrations of the targeted compounds, the smallest fractions of the observed effects were explained. In contrast to the chemical analysis, the measured effects in the bioassays (Effect and Toxic Units) for O.o.1 were not higher than for the other animals.^41^*Vice versa*, for animals with lower compound concentrations, like P.p.3 and P.p.5, the specific and non-specific effects measured in the three bioassays were similar to the extracts from other animals. This is an unexpected observation, as especially the cytotoxic effects were rather non-specific and could be caused by numerous chemicals. Our results demonstrate that a large fraction of unknown chemicals (and/or compounds that were not targeted or were below the limit of detection of chemical analysis) contributed substantially to the activation of AhR and PPARγ, receptors that are important for xenobiotic metabolism, the Nrf2-dependent oxidative stress response, as well as cytotoxicity, which integrates all cellular toxicity pathways into one apical outcome. The bioanalytical approach allows the inclusion of unknown and/or unexplained chemicals causing adverse effects in the risk assessment. Combining both data sets using the iceberg model underlines that chemical analysis and bioassays are complementary to comprehensively characterize the mixture exposome.

## 5 Conclusion

The results from chemical analysis have shown that compounds banned years to decades ago (*e.g.* PCBs) can still be highly relevant in the environment since they continue to be found in high concentrations in marine mammals, partly exceeding known threshold values of physiological impacts. Nonetheless, including these contaminants into iceberg modeling to explain measured cellular effects, in most cases only small fractions could be accounted for. This discrepancy indicates that there are still a lot of unknown (or untargeted) chemicals, activating toxicity pathways, supporting and clarifying previous findings.^33^

Besides the high contaminant burden of marine mammals demonstrated in this study, other reports from the literature show that these animals are under severe health stress and at high risk of dying from infectious diseases.^3,11,29,86–89^ In addition to adult males, which do not transfer part of their HOCs body burden to their offspring, especially young marine mammals (most of the individuals analyzed in this study fall into this category) are exposed to high concentrations of HOCs due to maternal transfer of HOCs during pregnancy and lactation, potentially causing physiological effects such as immunosuppression.^11^ The measurement and monitoring of the chemical burden by quantification of legacy compounds as well as emerging compounds with similar properties helps to strengthen the link between exposure and adverse health outcomes for the organisms and the ecosystem.

## Author contributions

EBR: conceptualization, data curation, formal analysis, investigation, methodology, validation, visualization, writing – original draft; BIE: formal analysis, methodology, writing – review & editing; ERN: formal analysis, investigation, validation, writing – review & editing; HN: investigation, validation, writing – review & editing; US: resources, writing – review & editing; AJ: conceptualization, investigation, methodology, project administration, supervision, validation, funding acquisition, writing – review & editing.

## Conflicts of interest

There are no conflicts to declare.

## Supplementary Material

EM-025-D3EM00033H-s001

EM-025-D3EM00033H-s002
