# Supplementary material for: Characterizing the marine mammal exposome by iceberg modeling, linking chemical analysis and in vitro bioassays
Source: Environ Sci Process Impacts. 2023 May 3;25(11):1802–16. doi: 10.1039/d3em00033h (PMC10647987; doi:10.1039/d3em00033h)
Supplement: EM-025-D3EM00033H-s001 [file EM-025-D3EM00033H-s001.pdf]

Electronic Supplementary Material (ESI) for Environmental Science: Processes & Impacts.
This journal is © The Royal Society of Chemistry 2023

Electronic Supplementary Information (ESI)

Characterizing the marine mammal exposome by iceberg modeling, linking chemical analysis and *in vitro* bioassays

Eva B. Reiter[a], Beate I. Escher[b,c], Elisa Rojo-Nieto[a], Hannah Nolte[a,d], Ursula Siebert[e] and Annika Jahnke[a,d]

[a] Department of Ecological Chemistry, Helmholtz Centre for Environmental Research - UFZ, Permoserstr. 15, 04318 Leipzig, Germany

[b] Department of Cell Toxicology, Helmholtz Centre for Environmental Research - UFZ, Permoserstr. 15, 04318 Leipzig, Germany

[c] Environmental Toxicology, Department of Geosciences, Eberhard Karls University Tübingen, Schnarrenbergstr. 94-96, 72076 Tübingen, Germany

[d] Institute for Environmental Research, RWTH Aachen University, 52074 Aachen, Germany

[e] Institute for Terrestrial and Aquatic Wildlife Research, University of Veterinary Medicine Hannover, Foundation, Werftstr. 6, 25761, Büsum, Germany

ESI Text

Text S1. Determination of the lipid content S3

Text S2. Cleanup procedure for GC-HRMS extracts S3

Text S3. GC-HRMS instrumental set up S4

Text S4. QA/QC chemical analysis S5

Text S5. Conversion of silicone-based concentrations c_{PDMS} to lipid-based concentrations $c_{lipid,eq.}$ S6

Text S6. Bioanalytical analysis and Iceberg Modeling S6

Text S7. Samples exceeding the maximal calibration concentration S7

ESI Figures

Figure S1. Relationship between the lipid content of the different organs and the lipid uptake into the PDMS, resulting in a weight gain, after PES S9

Figure S2. Concentration of the analyzed compound groups as total sum (∑) in the different tissues of the 12 individuals (Table S1). S10

Figure S3. Contaminant patterns of the analyzed compound groups as sum in the different tissues of the 12 individuals (Table S1) relative to the total chemical burden. S11

Figure S4. Heatmap showing the preferred accumulation of selected compounds in liver or blubber. S12

Figure S5. Lipid-normalized concentrations from solvent extraction (c_{lipid}) plotted against concentrations in the PDMS converted into lipid concentrations ($c_{lipid,eq.}$). S12

Figure S6. Sum concentration of the 13 PCBs (∑PCB13) of all measured animals and their according organs. S13

Figure S7. Medium-air partition constant $K_{medium/air}$ for detected chemicals in the marine mammal organs (Table S4). S14

Figure S8. Fraction of known (explained) effect data compared to the unknown effect data, normalized to the total concentration found within the extract. S15

Figure S9. Comparison of bioanalytically measured mixture effect with the predicted effect from the detected compounds in the PPARγ-bla bioassay S16

The additional MS Excel document contains ESI Table S1-S8:

Table S1. Further information on the twelve individuals analyzed.

Table S2. Details related to the marine mammal organs and PES.

Table S3. List of target chemicals, their compound categories, CAS number, chemical formula and the respective internal standards used for quantification.

Table S4. Chemicals detected in this study, their MDL (Method Detection Limits) and their physiochemical properties and the cytotoxicity (IC_{10}) and effect concentrations (EC_{10}) in the bioassays.

Table S5. Analytical results of the PDMS extracts in c_{PDMS} ($pg_{analyte}/mg_{PDMS}$).

Table S6. Analytical results of selected compounds of the solvent extracted blubber samples in c_{lipid} ($pg_{analyte}/mg_{lipid}$).

Table S7. Converted concentrations in PDMS to lipid-associated concentrations in $c_{lipid,eq.}$ ($pg_{analyte}/mg_{lipid}$).

Table S8. Iceberg modeling of effect in the AhR-CALUX, AREc32 and PPARγ-bla and cytotoxicity in the AhR-CALUX assay.

Text S1. Determination of the lipid content

The determination of the lipid fraction of the tissues was performed gravimetrically as described before,[1, 2] as a modified solvent extraction method described by Smedes et al.[3] In detail, 50 mg of homogenized blubber tissue and approximately 300 mg of homogenized liver, brain or kidney tissue were extracted in centrifuge tubes with 1.3 mL cyclohexane (Merck, GC-grade), 1 mL isopropanol (Merck, GC-grade) and 1.47 mL milliQ-water (Milli-Q Water Purification System from Merck-Millipore). For each sample, a duplicate measurement was carried out. The tubes were mixed for 30 s on a vortex mixer and centrifuged at 4000 rpm for 5 min. The upper cyclohexane phase was transferred with a glass pipette and collected in a pre-weighted vial. The extraction was repeated three times with 1.16 mL cyclohexane and 0.175 mL isopropanol each and the cyclohexane supernatant collected. The combined extracts were dried by using a gentle nitrogen stream and left in a desiccator overnight. The weight of the vial was determined with a micro-analytical balance (Mettler Toledo, Gießen, Germany). In addition, a negative control with solvent, another negative control with bovine serum albumin (Sigma-Aldrich) and a positive control with a triplicate sample of triolein (Sigma-Aldrich) were processed in parallel. The resulting weights were normalized by the mean recovery of the triolein samples.

Text S2. Cleanup procedure for GC-HRMS extracts

The cleanup procedure was performed as described before.[1] In detail, the extracts from the liver tissues of P.p.1 and P.p.2, the brain tissue of P.p.4 were submitted to freeze-out[4] in combination with a primary secondary amine (PSA) sorbent (Agilent Technologies, USA) extraction.[5] For the extract of the kidney from P.p.3, after freeze-out and PSA extraction, an additional cleanup with EMR-Lipid cartridges[4] (3 mL, Agilent Technologies, USA) was carried out. For the other extracts, the combination of EMR-Lipid cartridges and PSA extraction was applied. Ideally, all extracts would have been submitted to the same cleanup procedure. For the bioanalytical work, a freeze-out in combination with a PSA sorbent was conducted. Unfortunately, matrix residues after this cleanup interfered with the performance and lifetime of the GC-HRMS instrument. For instrumental analysis a cleanup combination of EMR-Lipid cartridges and PSA extraction performed the best, for optimal resolution of the analytical results. The other way around, extracts after an EMR-Lipid cleanup interfered with the bioanalytical measurements, i.e. the extracts of procedural blanks were causing cytotoxic effects. This is the reason why different cleanup procedures were conducted for the two (bio)analytical pipelines. In order to achieve an accurate quantification, a method-matched calibration was used that underwent the same cleanup procedures as the sample extracts.

In spite of different applied cleanup procedures, the chemical recoveries between EMR and freeze-out were similar, as described in Muz et al.[4] More precisely, for those chemicals detected and used for iceberg modeling in this study, an average recovery of 92 % (range: 80-99 %, n=25) for EMR was calculated, whereas for freeze-out an average recovery of 97 % (75-112 %, n=26) was determined. No recovery could be calculated for PCB 118 after EMR cleanup.[4] On average, recoveries after freeze-out were 1.07 times higher and thus differences due to the different cleanup procedures were considered to be negligible.

To carry out the freeze-out cleanup[4], the solvent of the sample extract was transferred into a vial with conical bottom and the solvent was exchanged from EtAc to 1.5 mL Acetonitrile (ACN, Merck, GC-grade) using a gentle nitrogen stream to evaporate the solvent. The sample extract in ACN was stored at -20 °C for at least 2 h, to allow the lipid residue to settle to the bottom of the vial. The supernatant was transferred carefully with a glass pipette. The freeze-out was repeated with another 1.5 mL aliquot of ACN and the supernatant transferred again after a minimum time of 2 h at -20 °C.

The EMR-Lipid cleanup[4] was carried out with 3 mL cartridges. For this workflow, the solvent of the sample extract was exchanged from EtAc to 1.5 mL ACN using a gentle nitrogen stream to evaporate the solvent. The ACN extract passed the EMR-Lipid cartridge and another 1.5 mL aliquot of ACN was loaded to the cartridge. A gentle pressure of less than 0.2 bar was applied to fully recover the extract after the cleanup.

The PSA extraction was applied after the freeze-out or EMR, to optimize the removal of disturbing matrix residues. The 3 mL extracts in ACN (after freeze-out or EMR) were transferred to a centrifuge tube filled with approximately 35 mg of PSA and 200 mg of dried magnesium sulfate. The extract vial was washed with 1 mL fresh ACN, and the solvent was also transferred to the centrifuge tube. The tube was mixed for 1 min with a vortex mixer and centrifuged for 5 min at 5000 rpm. The supernatant was transferred carefully with a glass pipette. The centrifuge tube was filled with another 4 mL aliquot of ACN and mixed and centrifuged with the same setup.

Text S3. GC-HRMS instrumental set up

To quantify the chemical concentration in the PDMS sheets, a gas chromatography-high resolution Orbitrap mass spectrometry (GC-HRMS) measurement was performed. A 13-point calibration, ranging from 0.1 to 1000 ng mL^{-1}, as well as solvent blanks and silicone blanks were prepared with the same method as the samples. Every batch that was run with the GC-HRMS contained the calibration and solvent blanks (prepared with the same cleanup procedure as the measured samples), 3-6 procedural silicone blanks and 12-18 samples. In addition, the 100 ng mL^{-1}-calibration point was injected in the middle of the batch and served as quality control, for the same reason the calibration concentrations 0.1, 1, 10, 100 and 1000 ng mL^{-1} were additionally measured at the end of every batch.

Separation of the 117 target chemicals was achieved with a GC system consisting of a TriPlus RSH autosampler with a Trace 1310 GC coupled with a Thermal Desorption Unit (TDU-2) and a Cooled Injection System (CIS, both from Gerstel, Mülheim, Germany). The injections were made in splitless mode with an injection volume of 2 µL. Helium was used as carrier gas at a constant flow of 1.2 mL/min. The thermal desorption in the TDU was carried out with the heating program from 30 °C to 300 °C at a heating rate of 300 °C/min (holding 5 min). The transfer temperature of the TDU on the top of the CIS was set at 320 °C. After refocusing in the glass liner with deactivated glass wool (CIS-4 TDU, Gerstel, Mülheim, Germany) at -25 °C for 0.2 min, the analytes were desorbed with a temperature of up to 300 °C at a fast rate of 12 °C/s and a final holding time of 10 min and injected in a splitless mode with a time of 2 min. The chromatographic separation was based on the following temperature program: 60 °C (1 min), up to 150 °C at 30 °C /min, up to 186 °C at 6 °C/min rate, up to 300 °C with a rate of 4 °C/min (holding 11.5 min). The GC

was coupled with a QExactive instrument (Thermo Fisher Scientific, Germany) via a transfer line kept at 280 °C. The ion source temperature was set at 250 °C. Mass spectrometric analysis was performed using electron ionization (EI) at 70 eV in positive polarity, in full-scan mode with a scan range of 70-810 *m/z* and a resolution of 60,000 (FWHM at *m/z* 200). The internal calibration and tuning of the instrument were established using Perfluorotributylamine (PFTBA) as a mass calibrant. The data was processed and peak areas were integrated using the software Tracefinder General Quan 5.1 (Thermo Fisher Scientific). The quantification of the target compounds was based on an internal standard calibration method. 21 isotope-labeled compounds were used, selected according to their retention time and chemical family to calculate area ratios to the target compounds (Table S3).

Text S4. QA/QC chemical analysis

For the GC-HRMS maintenance a long-term record was available and evaluation of tuning and calibration (with PFTBA) of the instrument was performed before every sequence. Method Detection Limits (MDLs) were determined for the sampling method (see below). Quantification of the target compounds was performed using a 13-point method-matched calibration with a concentration range between 0.1 to 1000 ng mL^{-1}. In this study, QA/QC of the samples was carried out running replicates (n=2-6, see Table S2). In general, is recommended that at least 10 % of the samples are analyzed in duplicates, and thus the study fulfills this requirement. A quality control sample (a calibration sample of 100 ng mL^{-1} after a predefined number of samples) was run with every sequence, to check for cross contamination and a possible decline in instrument performance, as well as allowing to evaluate potential shifts in the retention times. In addition, selected calibration points (0.1, 1, 10, 100 and 1000 ng mL^{-1}) were measured at the end of the sequence of every batch. The sequences included instrument blanks, running at the beginning, following every two to three sample injections and in the end of the sequence. Together with the calibration, solvent blanks, PDMS procedural blanks and the quality control samples were run with every batch of 12-18 samples.

The positive identification of the compounds was based on the presence of the isotope "quantifier" *m/z* and two additional fragments *m/z* to confirm the compound ("qualifiers") at a given retention time.

Method detection limits (MDL) were applied, according to the values by Rojo-Nieto et al.[6], Table S4. These MDLs were estimated using a two-tailed t-distribution test with 99 % interval, based on the US EPA guidelines.[7] Those were calculated with matrix-matched extracts, i.e. using 1 g of silicone equivalent extract and the co-extracted lipids from a sample of liver cod oil and salmon oil 50/50 (nominally 10 mg lipids), spiked with standard solutions at 1, 10 and 100 ng mL^{-1} (n=4 at each level) and submitted to ERM-Lipid cartridges and PSA cleanup. For 14 compounds, no MDL was available and values were taken from Muz et al.[4], in this case prepared with 10 mg pork lipid extract and 1 g silicone equivalent extract and submitted to the ERM-Lipid cleanup. The MDL was applied before silicone- or lipid-normalization and thus the unit differs from the reported results.

Due to lipid uptake to the PDMS, the concentration of the extracts can be described as $n_{PDMS+co\text{-}extracted\ lipids}$ (see Equation S1). For chemical analysis, a lipid-correction was performed, by subtracting the fraction of the co-extracted lipid, using the $K_{Lipid/PDMS}$ values in Table S4 (Equation S2 and S3).

$$n_{extract}=n_{PDMS+co\text{-}extracted\ lipid}= n_{PDMS}+ n_{co\text{-}extracted\ lipid} \quad \text{(Equation S1)}$$

$$n_{PDMS}=n_{extract}*(K_{lipid/PDMS}* m_{lipid}*m_{PDMS}^{-1})^{-1} \quad \text{(Equation S2)}$$

$$K_{lipid/PDMS} = c_{lipid}* c_{PDMS}^{-1} \quad \text{(Equation S3)}$$

Text S5. Conversion of silicone-based concentrations c_{PDMS} to lipid-based concentrations $c_{lipid,eq.}$

In order to confirm that the converted values using the partition coefficients were appropriate, we compared the converted $c_{lipid,eq.}$ values, using experimentally determined $K_{lipid/PDMS}$, with our measured c_{lipid} values from total exhaustive extraction (Table S6). In Figure S5, the $c_{lipid,eq.}$ and c_{lipid} values detected in the blubber samples from the seven individuals P.p.1-5, P.v.1 and O.o.1 are plotted together. Only compound groups of 13 PCBs, 8 PAHs, 8 OCPs and 2 CHCs, for which experimentally determined $K_{lipid/PDMS}$ values are available, were evaluated and plotted. In general, PCBs, OCPs and CHCs were in good agreement and appeared to be in a 2:1 to 1:1 range. However, for PAHs the converted $c_{lipid,eq.}$ values were systematically smaller than c_{lipid}, from solvent extraction, by a factor of 10±12 (mean±SD, *n*=26). The equilibration process between blubber and PDMS was performed for 48-72 h at 4 °C with no apparent tissue decay and thus metabolic effects caused by microorganisms are unlikely. Due to this observed discrepancy, PAHs have been excluded from the comparison with other studies using the lipid-based concentrations. The $c_{lipid,eq.}$ values were calculated for all 70 compounds and listed in Table S7. Values that were translated with a $K_{lipid/PDMS}$ value from literature are marked accordingly.

Text S6. Bioanalytical testing and Iceberg Modeling

In our previous study,[1] PDMS extracts from the organs of P.p.1-5, P.v.1 and O.o.1 were measured in three cell-based *in vitro* bioassays,namely PPARγ-bla GeneBLAzer assay,[8] AhR-CALUX assay[9] and AREc32 assay.[10] The measured toxic actions were expressed as Effect Units (EU_{bio}), which is the reciprocal of the effect concentration causing 10 % of the specific effect relative to a reference compound (EC_{10}, PPARγ-bla and AhR-CALUX) and the effect concentration triggering an induction ratio of 1.5 ($EC_{IR1.5}$, for AREc32). Furthermore, the Toxic Unit (TU_{bio}) was determined, as the reciprocal of the inhibitory concentration causing a 10 % reduction of cell viability (IC_{10}), which indicates nonspecific cytotoxic effects.

For effect modeling, the bioanalytical equivalent concentrations (BEQ_{bio}) were calculated from the EC values of the sample and a reference compound: Rosiglitazone for PPARγ-bla and Benzo[a]pyrene for AhR-CALUX and AREc32 (see Table S8), according to Equation S4. The corresponding predicted BEQ_{chem} of the sample extract was calculated by the sum of the single $BEQ_{chem}(i)$ of all detected compounds *i*, which are defined as their relative effect potencies REP(*i*) multiplied by the concentration c(*i*) (see Equation S5 and

S6). The ratio of BEQ_{chem}/BEQ_{bio} indicates which fraction of the measured effect in the bioassay can be explained by the detected compounds.

$$BEQ_{bio} = \frac{EC_{10}(\text{reference})}{EC_{10}(\text{sample})} \text{ or } \frac{EC_{IR1.5}(\text{reference})}{EC_{IR1.5}(\text{sample})} \quad \text{(Equation S4)}$$

$$BEQ_{chem} = \sum_{i=1}^{n} BEQ_{chem}(i) = \sum_{i=1}^{n} (REP(i)*c(i)) \quad \text{(Equation S5)}$$

$$REP(i) = \frac{EC_{10}(\text{reference})}{EC_{10}(i)} \text{ or } \frac{EC_{IR1.5}(\text{reference})}{EC_{IR1.5}(i)} \quad \text{(Equation S6)}$$

In addition, the iceberg model was applied to the cytotoxic effects by the ratio TU_{chem}/TU_{bio}, for which TU_{chem} was calculated from the cytotoxic effect from the mixture of the detected compounds (Equation S7). TU_{bio} was calculated earlier.[1]

$$TU_{chem} = \sum_{i=1}^{n} TU_{chem}(i) = \sum_{i=1}^{n} \frac{c(i)}{IC_{10}(i)} \quad \text{(Equation S7)}$$

All EC(*i*) and IC(*i*) values used for iceberg modeling in this study were taken from previous measurements[11, 12] or the Tox21 database,[13] as listed in Table S4.

The extracts submitted to the bioassays were extracted from 350 µm PDMS sheets which had been submitted to a freeze-out cleanup combined with a PSA extraction. As the extracts submitted to the bioassays were not corrected for co-extracted lipids, no lipid correction of the predicted mixture effect BEQ_{chem} was performed and furthermore, only the average concentrations from the two 350 µm PDMS sheet replicates were used to calculate the BEQ_{chem}.

Text S7. Samples exceeding the maximal calibration concentration

The concentration of a few chemicals found in some extracts were above the maximal concentration of the calibration, e.g. up to a factor of 4 for the following: PCBs 138, 149, 153, 4,4'-DDE, Dieldrin and Galaxolide in some P.p.1 liver extracts; PCB 153 in some P.p.2 liver extracts; PCB153 in P.v.2 in the extracts of all organs and PCB138 in brain and kidney extracts of P.v.2. However, the highest concentrations were found in the extracts of O.o.1; the following compounds exceeded the maximal concentration of the calibration in one or more organs: PCBs 52, 101, 118, 138, 149, 153, 170, 4,4'-DDE, Dieldrin Ethylene glycol diphenylether. As an example, for PCB 153 the sample extracts of O.o.1 liver was up to a factor of 15 above the maximal concentration of the calibration. Sample extracts that exceeded the maximal concentrations from the calibration are highlighted accordingly in Table S5.

For every tissue from O.o.1 six replicates were available, as duplicates of three different PDMS thicknesses were sampled. The thinnest PDMS slides (125 µm) exceeded the highest calibration between a factor of 1.3 to 5.6 (for PCBs 52, 138, 149 and 153). The relative standard deviation of the six replicates for the PCBs ranged between 1.5 and 22 % and thus the average concentration can be expected to be precise, although the calibration maximum was exceeded. A repeated instrumental analysis was not performed, as the low extract volume of the samples did not allow for repeated injections. Since the quantification method was based on relative response factors that were derived from the peak area of the native analyte normalized

to the peak area of the associated isotope-labeled standard, a dilution of the extract or injection of a smaller extract volume does not change the ratio. Inclusion of a two orders of magnitude higher concentrated calibration extract into the instrumental analysis is not recommended as it can cause instrumental issues due to e.g. column overloading and detector saturation. Furthermore, due to limited sample size, the extraction of the biota samples could not be repeated. Hence, an extrapolation of the linear calibration curve was performed. The PCB concentration of blubber from O.o.1 was analyzed before by Schnitzler et al.[14] who reported an around 4 times higher PCB burden in this individuum. This is discussed in chapter "3.4 Integration of chemical data to previously measured data from literature" (main manuscript). More importantly, if only the PCB congeners are compared that did not exceed the maximal concentration of the calibration, the results still differ by a factor of 4. As the ratio does not differ between congeners that exceeded the maximal concentration of the calibration, we assume the concentrations evaluated in this study to still be correct, and still in the linear phase of the calibration curve, though extrapolated.

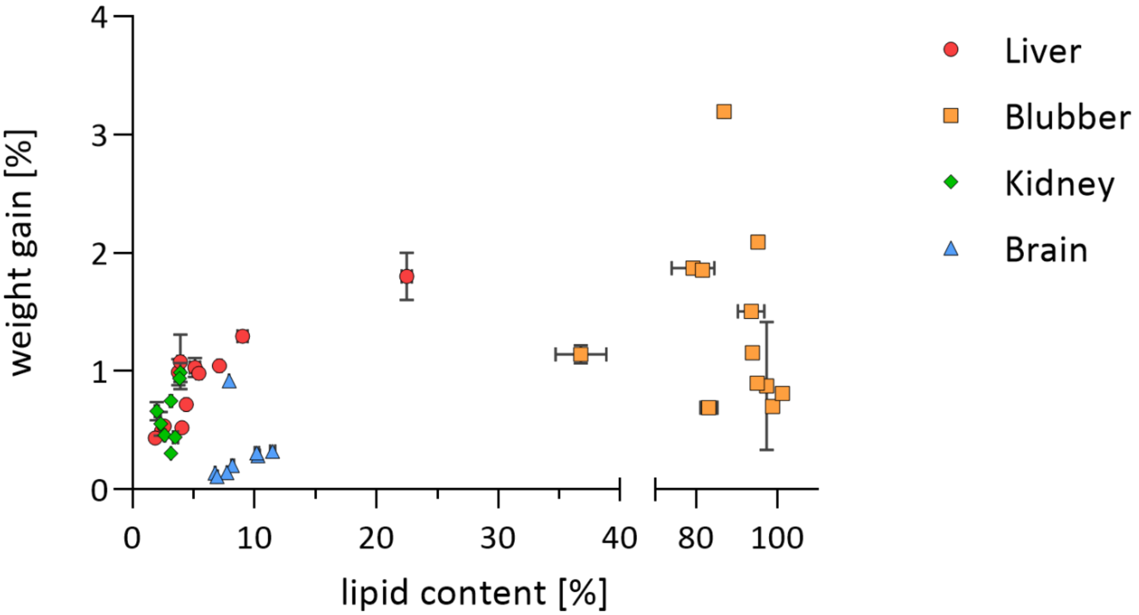

Figure S1. Relationship between the lipid content of the different organs and the lipid uptake into the PDMS, resulting in a weight gain, after chemometer sampling. The points represent the mean values and the bar the SD.

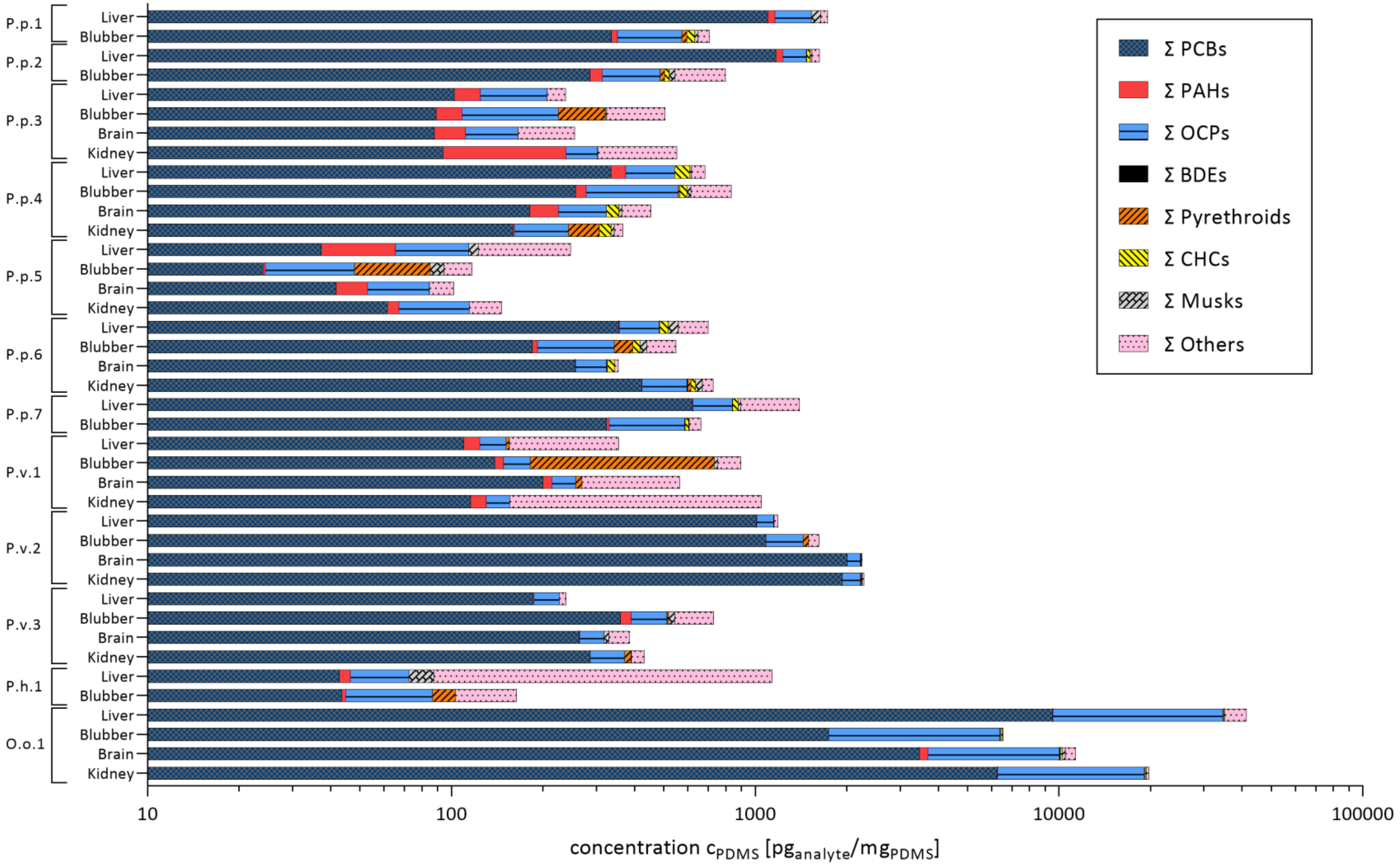

Figure S2. Concentrations of the analyzed compound groups as total sum (∑) in the different tissues of the 12 individuals (Table S1). P.p.: harbor porpoise, P.v.: harbor seal, P.h.: ringed seal, O.o.: orca, plus running number. PCBs: polychlorinated biphenyl, PAHs: Polycyclic Aromatic Hydrocarbons, OCPs: Organochlorine Pesticides, BDEs: (Poly)brominated Diphenyl Ethers, CHCs: Chlorinated Hydrocarbons. See Table S3 for full target compound list and Table S5 for the single concentrations. To addition to this bar chart, Figure S3 displays the contaminant pattern normalized to the total chemical burden.

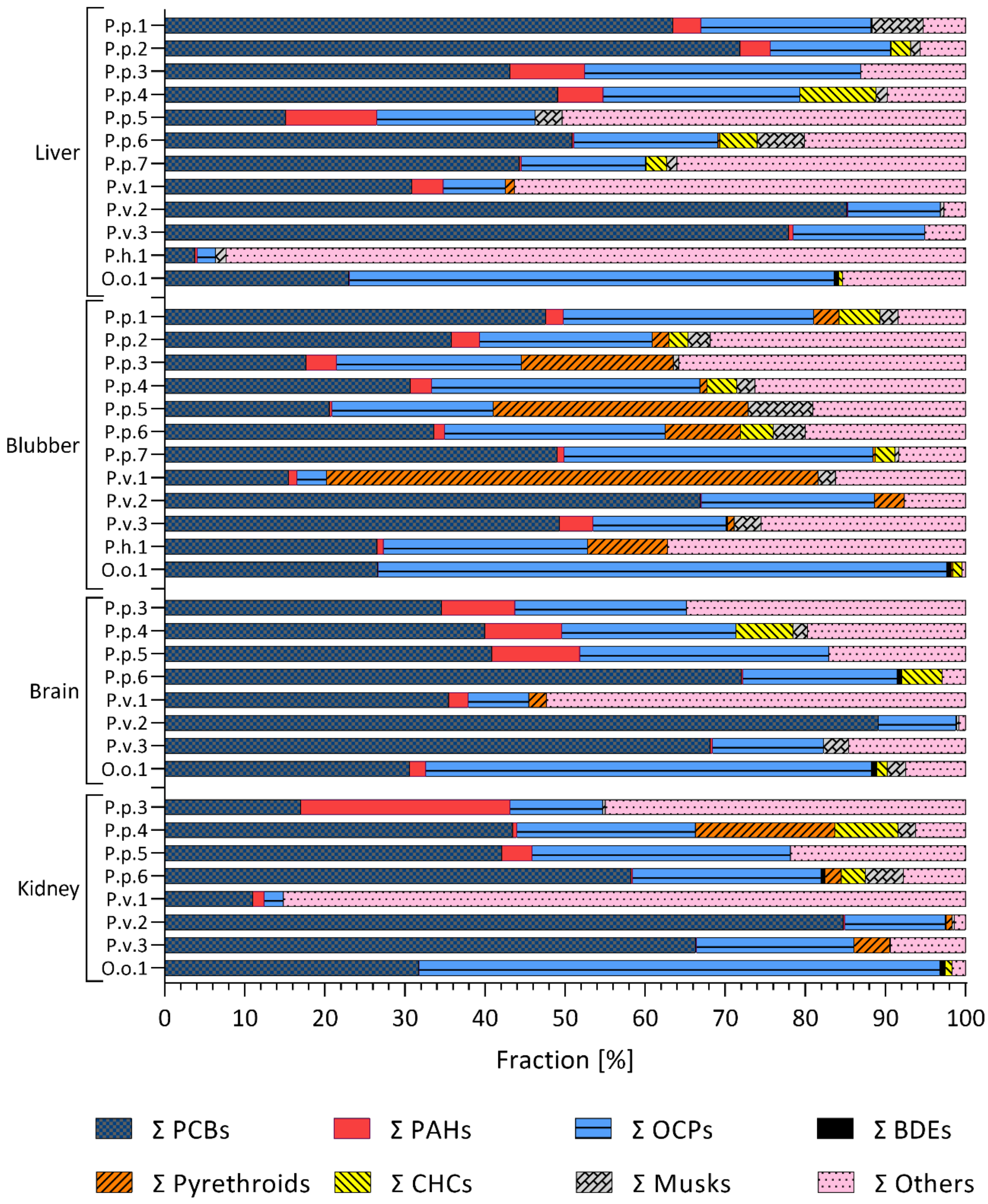

Figure S3. Contaminant patterns of the analyzed compound groups as sum (∑) in the different tissues of the 12 individuals (Table S1) relative to the total chemical burden. P.p.: harbor porpoise, P.v.: harbor seal, P.h.: ringed seal, O.o.: orca, plus running number. PCBs: polychlorinated biphenyls, PAHs: Polycyclic Aromatic Hydrocarbons, OCPs: Organochlorine Pesticides, BDEs: (Poly)brominated Diphenyl Ethers, CHCs: Chlorinated Hydrocarbons. See Table S3 for full target compound list. To addition to this bar chart, Figure S2 displays the total chemical burden.

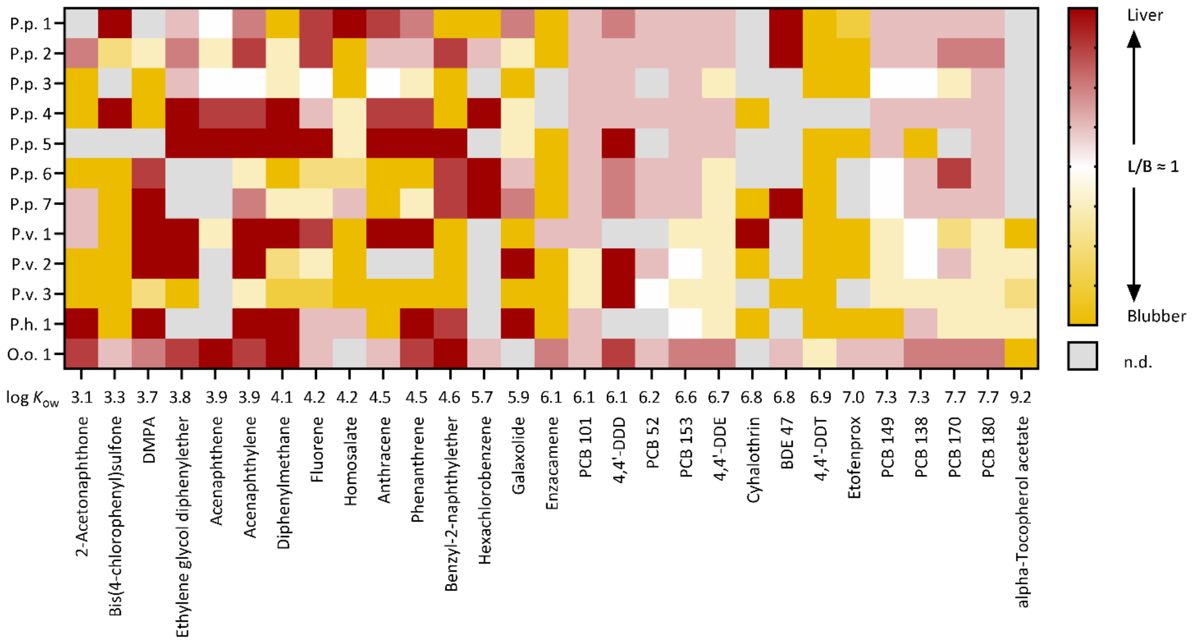

Figure S4. Heatmap showing the preferred accumulation of selected compounds in liver (red) or blubber (yellow). Compounds are sorted by increasing log K_{ow} values (left to right). Liver/blubber (L/B) activity ratios were calculated. For a L/B activity ratio of around 1 (i.e. 0.9-1.1), no preference was assumed (white color). If the compound was neither detected in liver nor blubber, it is colored in grey. P.p.: harbor porpoise, P.v.: harbor seal, P.h.: ringed seal, O.o.: orca, plus running number.

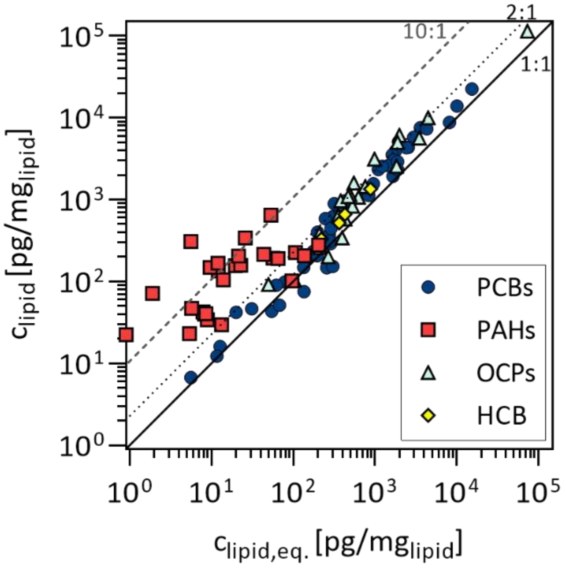

Figure S5. Lipid-normalized concentrations from solvent extraction (c_{lipid}) plotted against concentrations in the PDMS converted into lipid concentrations ($c_{lipid,eq.}$) in $pg_{compound}/mg_{lipid}$ from blubber extracts of P.p.1-5, P.v.1 and O.o.1, on a logarithmic scale. The solid line indicates the 1:1 fit, the dotted line the 2:1 fit and the grey dashed line the 10:1 fit relationship.

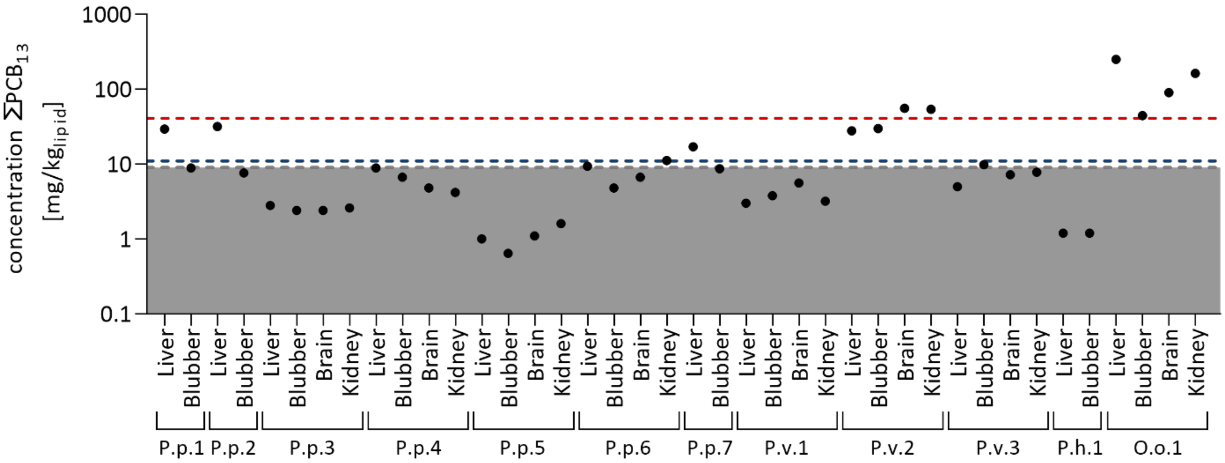

Figure S6. Sum concentration of the 13 PCBs (∑PCB_{13}) of all measured animals and their according organs: P.p.: harbor porpoise, P.v.: harbor seal, P.h.: ringed seal, O.o.: orca, plus running number. Additionally, ∑PCB thresholds from literature are marked: grey dashed line at 9.0 mg/kg_{lipid} (∑PCB_{23}) for general physiological impacts in marine mammals,[15, 16] blue dashed line at 11.0 mg/kg_{lipid} (∑PCB_{25}) for infertility/reproductive failure expected in female sexually mature harbor porpoises,[17, 18] and red dashed line at 41.0 mg/kg_{lipid} (∑PCB_{23}) for profound reproductive impairment of Baltic ringed seals.[19, 20]

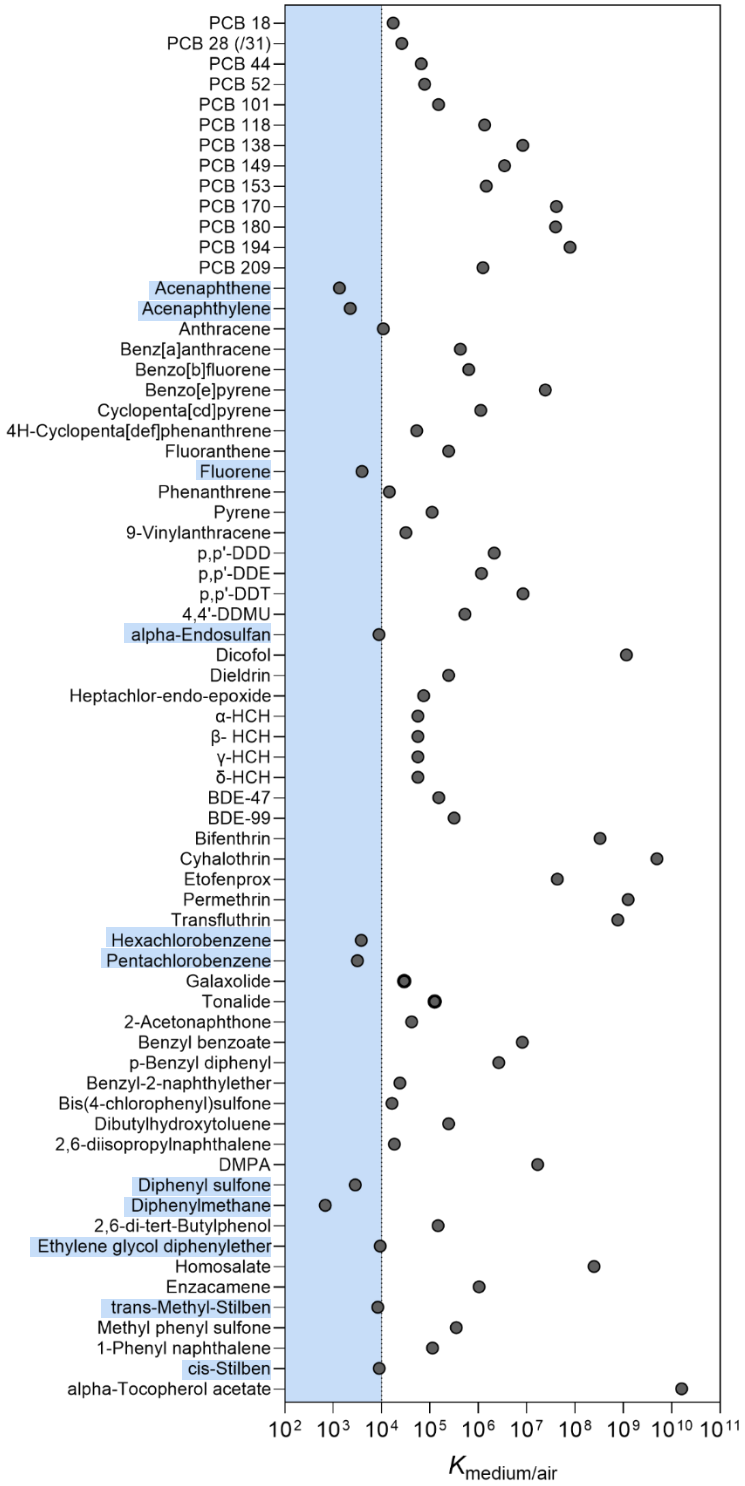

Figure S7. Medium-air partition constant $K_{\text{medium/air}}$ for detected chemicals in the marine mammal organs (Table S4). The dotted line marks the volatility cutoff ($K_{\text{medium/air}} = 10^4$) and chemicals with $K_{\text{medium/air}} < 10^4$ (the blue shaded area) are expected not to be measurable in the bioassay system, due to partitioning into the headspace of the plate-based bioassay despite being sealed and fitted with a cover.[13] However, effect data for alpha-Endosulfan and Ethylene glycol diphenylether are available.

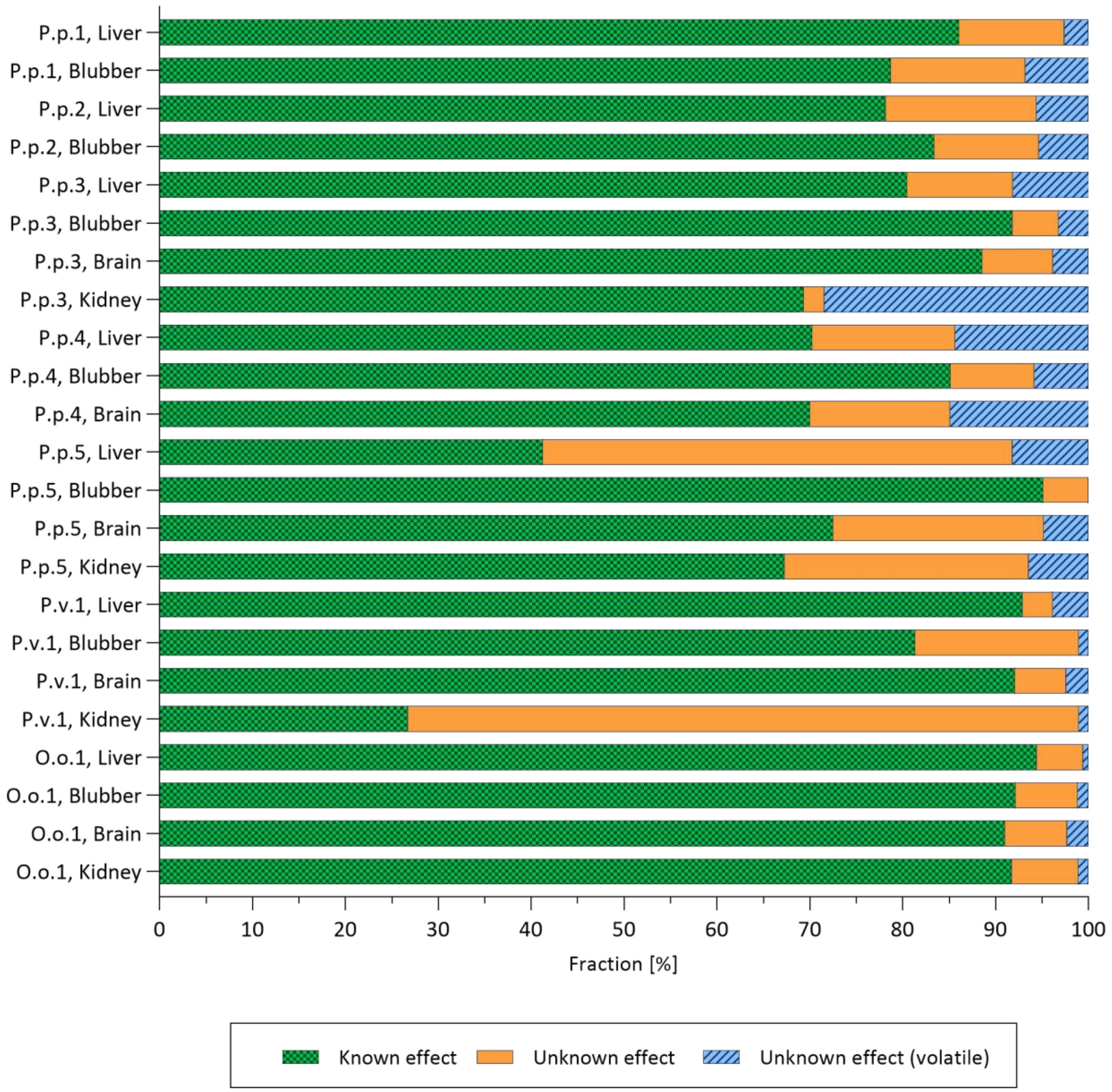

Figure S8. Fractions of known (explained) effect data compared to the unknown effect data, normalized to the total concentrations found within the extract. From detected 70 compounds, for 37 compounds known effect data was available (green bar). For 24 compounds no effect data was available (orange bar) and for 9 chemicals the effect data was not available as they are expected to be too volatile in the bioassay system (blue bar). Listed for the samples that were bioanalytically analyzed (P.p.: harbor porpoise, P.v.: harbor seal, P.h.: ringed seal, O.o.: orca, plus running number).

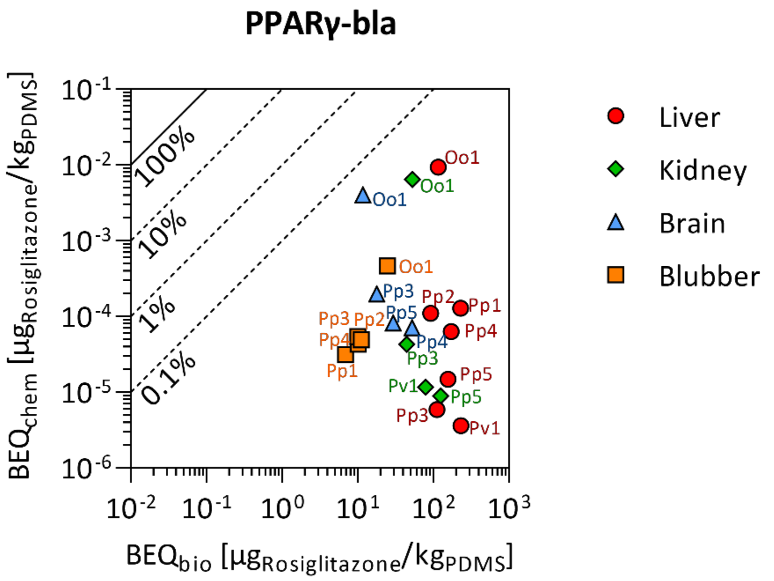

Figure S9. Comparison of bioanalytically measured mixture effects, expressed as bioanalytical equivalent concentrations (BEQ_{bio}) with the predicted effect from the detected compounds (BEQ_{chem}) in the PPARγ-bla bioassay for the samples from seven marine mammals (P.p.: harbor porpoise, P.v.: harbor seal, P.h.: ringed seal, O.o.: orca, plus running number).

ESI References

1. E. B. Reiter, B. I. Escher, U. Siebert and A. Jahnke, Activation of the xenobiotic metabolism and oxidative stress response by mixtures of organic pollutants extracted with in-tissue passive sampling from liver, kidney, brain and blubber of marine mammals, *Environ Int*, 2022, **165**, 107337.
2. A. Baumer, B. I. Escher, J. Landmann and N. Ulrich, Direct sample introduction GC-MS/MS for quantification of organic chemicals in mammalian tissues and blood extracted with polymers without clean-up, *Anal Bioanal Chem*, 2020, **412**, 7295-7305.
3. F. Smedes, Determination of total lipid using non-chlorinated solvents, *Analyst*, 1999, **124**, 1711-1718.
4. M. Muz, E. Rojo-Nieto and A. Jahnke, Removing Disturbing Matrix Constituents from Biota Extracts from Total Extraction and Silicone-Based Passive Sampling, *Environ Toxicol Chem*, 2021, **40**, 2693-2704.
5. T. Wernicke, S. Abel, B. I. Escher, J. Koschorreck, H. Rüdel and A. Jahnke, Equilibrium sampling of suspended particulate matter as a universal proxy for fish and mussel monitoring, *Ecotoxicol Environ Saf*, 2022, **232**, 113285.
6. E. Rojo-Nieto, M. Muz, J. Watzke and A. Jahnke, Using chemometers to understand the thermodynamics of bioaccumulation in aquatic environments (in prep.).
7. U. S. EPA, Title 40: Protection of Environment; Part 136-Guidelines Establishing Test Procedures for the Analysis of Pollutants; Appendix B to Part 136-Definition and Procedure for the Determination of the Method Detection Limit-Revision 1.11.*Journal*, 2011.
8. Invitrogen, GeneBLAzer® PPAR Gamma 293H DA and PPAR Gamma-UAS-bla 293H Cell-based Assay Protocol. Invitrogen Corporation, Carlsbad, CA, USA., 2010.
9. P. A. Neale, R. Altenburger, S. Aït-Aïssa, F. Brion, W. Busch, G. de Aragao Umbuzeiro, M. S. Denison, D. Du Pasquier, K. Hilscherová, H. Hollert, D. A. Morales, J. Novák, R. Schlichting, T. B. Seiler, H. Serra, Y. Shao, A. J. Tindall, K. E. Tollefsen, T. D. Williams and B. I. Escher, Development of a bioanalytical test battery for water quality monitoring: Fingerprinting identified micropollutants and their contribution to effects in surface water, *Water Res*, 2017, **123**, 734-750.
10. B. I. Escher, M. Dutt, E. Maylin, J. Y. Tang, S. Toze, C. R. Wolf and M. Lang, Water quality assessment using the AREc32 reporter gene assay indicative of the oxidative stress response pathway, *J Environ Monit*, 2012, **14**, 2877-2885.
11. J. Lee, G. Braun, L. Henneberger, M. Konig, R. Schlichting, S. Scholz and B. I. Escher, Critical Membrane Concentration and Mass-Balance Model to Identify Baseline Cytotoxicity of Hydrophobic and Ionizable Organic Chemicals in Mammalian Cell Lines, *Chemical research in toxicology*, 2021, **34**, 2100-2109.
12. A. Baumer, S. Jäsch, N. Ulrich, I. Bechmann, J. Landmann, A. Stover and B. I. Escher, Chemical mixtures in human post-mortem tissues assessed by a combination of chemical analysis and in vitro bioassays after extraction with silicone, *Environ Int*, 2021, **157**, 106867.
13. B. I. Escher, L. Henneberger, M. Konig, R. Schlichting and F. C. Fischer, Cytotoxicity Burst? Differentiating Specific from Nonspecific Effects in Tox21 in Vitro Reporter Gene Assays, *Environ Health Perspect*, 2020, **128**, 77007.
14. J. G. Schnitzler, M. Pinzone, M. Autenrieth, A. van Neer, I. J. LL, J. L. Barber, R. Deaville, P. Jepson, A. Brownlow, T. Schaffeld, J. P. Thome, R. Tiedemann, K. Das and U. Siebert, Inter-individual differences in contamination profiles as tracer of social group association in stranded sperm whales, *Sci Rep*, 2018, **8**, 10958.
15. K. Kannan, A. L. Blankenship, P. D. Jones and J. P. Giesy, Toxicity Reference Values for the Toxic Effects of Polychlorinated Biphenyls to Aquatic Mammals, *Hum Ecol Risk Assess*, 2000, **6**, 181-201.

16. C. W. Tubbs, California condors and DDT: Examining the effects of endocrine disrupting chemicals in a critically endangered species, *Endocrine Disruptors*, 2016, **4**, e1173766.
17. M. J. van den Heuvel-Greve, A. M. van den Brink, M. J. J. Kotterman, C. Kwadijk, S. C. V. Geelhoed, S. Murphy, J. van den Broek, H. Heesterbeek, A. Grone and I. J. LL, Polluted porpoises: Generational transfer of organic contaminants in harbour porpoises from the southern North Sea, *Sci Total Environ*, 2021, **796**, 148936.
18. S. Murphy, J. L. Barber, J. A. Learmonth, F. L. Read, R. Deaville, M. W. Perkins, A. Brownlow, N. Davison, R. Penrose, G. J. Pierce, R. J. Law and P. D. Jepson, Reproductive Failure in UK Harbour Porpoises Phocoena phocoena: Legacy of Pollutant Exposure?, *PLoS One*, 2015, **10**, e0131085.
19. P. D. Jepson, R. Deaville, J. L. Barber, A. Aguilar, A. Borrell, S. Murphy, J. Barry, A. Brownlow, J. Barnett, S. Berrow, A. A. Cunningham, N. J. Davison, M. Ten Doeschate, R. Esteban, M. Ferreira, A. D. Foote, T. Genov, J. Gimenez, J. Loveridge, A. Llavona, V. Martin, D. L. Maxwell, A. Papachlimitzou, R. Penrose, M. W. Perkins, B. Smith, R. de Stephanis, N. Tregenza, P. Verborgh, A. Fernandez and R. J. Law, PCB pollution continues to impact populations of orcas and other dolphins in European waters, *Sci Rep*, 2016, **6**, 18573.
20. E. Helle, M. Olsson and S. Jensen, PCB levels correlated with pathological changes in seal uteri, *Ambio*, 1976, DOI: http://www.jstor.org/stable/4312230, 261-262.
